# Cell-type-specific responses to the microbiota across all tissues of the larval zebrafish

**DOI:** 10.1016/j.celrep.2023.112095

**Published:** 2023-02-13

**Authors:** Michelle S. Massaquoi, Garth L. Kong, Daisy Chilin-Fuentes, Julia S. Ngo, Patrick F. Horve, Ellie Melancon, M. Kristina Hamilton, Judith S. Eisen, Karen Guillemin

**Affiliations:** 1 Institute of Molecular Biology, University of Oregon, 1318 Franklin Boulevard, Eugene, OR 97403, USA; 2 Institute of Neuroscience, University of Oregon, 1254 University of Oregon, Eugene, OR 97403, USA; 3 Humans and the Microbiome Program, CIFAR, Toronto, ON M5G 1M1, Canada; 4 Thermo Fisher Scientific, 29851 Willow Creek Road, Eugene, OR 97402, USA; 5 Thermo Fisher Scientific, 22025 20th Avenue SE, Bothell, WA 98021, USA; 6 Lead contact

## Abstract

Animal development proceeds in the presence of intimate microbial associations, but the extent to which different host cells across the body respond to resident microbes remains to be fully explored. Using the vertebrate model organism, the larval zebrafish, we assessed transcriptional responses to the microbiota across the entire body at single-cell resolution. We find that cell types across the body, not limited to tissues at host-microbe interfaces, respond to the microbiota. Responses are cell-type-specific, but across many tissues the microbiota enhances cell proliferation, increases metabolism, and stimulates a diversity of cellular activities, revealing roles for the microbiota in promoting developmental plasticity. This work provides a resource for exploring transcriptional responses to the microbiota across all cell types of the vertebrate body and generating new hypotheses about the interactions between vertebrate hosts and their microbiota.

## In brief

The Larval Zebrafish Gnotobiotic Atlas provides a comprehensive resource of microbiota-dependent transcriptomes across the entire body at single-cell resolution. Massaquoi et al. show gene expression changes between conventionalized and germ-free cell types to the microbiota, not limited to tissues in direct contact with resident microbes.

## Graphical Abstract

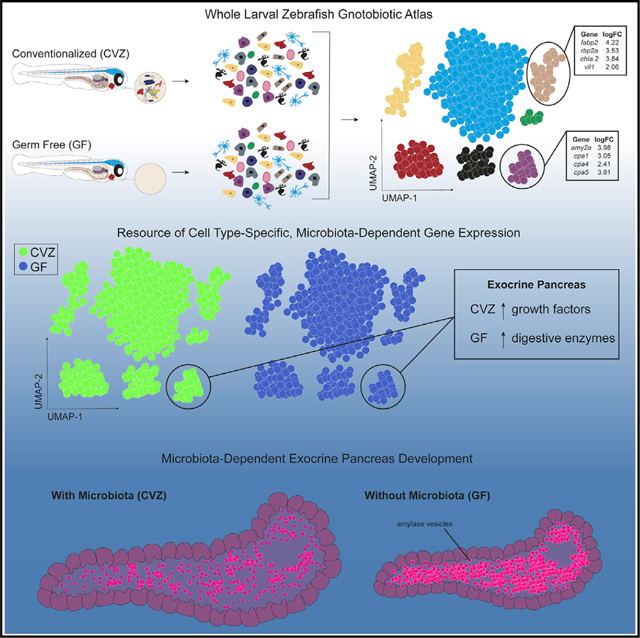

## INTRODUCTION

Bacteria were the first organisms to evolve on earth, preceding the emergence of animals by roughly 3 billion years. All animals evolved amidst the pressures exerted by the abundant bacteria, fungi, and viruses in their world, which necessitated cellular and tissue strategies to co-exist with resident microbial communities, or microbiota, that live on and within animal bodies. Emerging insights into the many roles that microbiotas play in host development and homeostasis are redefining how we view animal biology.^1^ The study of animal-microbiota interactions are being transformed by new technologies for single-cell characterizations,^2^ but the impacts of resident microbes on cellular transcription have not yet been described globally across an entire vertebrate animal body. The goal of this research is to create a resource that catalogs cell-type-specific transcriptional responses of the larval zebrafish to its microbiota. Zebrafish are an excellent model in which to study impacts of the microbiota on vertebrate biology because they are readily amendable to gnotobiotic manipulations,^3^ their small size allows single-cell transcriptional profiling of the entire body,^4^ and they share common developmental and physiological programs with larger vertebrates such as humans. In this study, we used the 10x Genomics pipeline to interrogate the transcriptomes of single cells dissociated from cohorts of larval zebrafish reared in the presence or absence of their normal microbiota. We report our analysis of the global, tissue, and cell type responses to the microbiota that emerge from this dataset, which will serve as a valuable hypothesis generating resource for the microbiome sciences field.

## RESULTS AND DISCUSSION

### Responsiveness to microbes is widespread across cells of the vertebrate body

**Figure 1.**
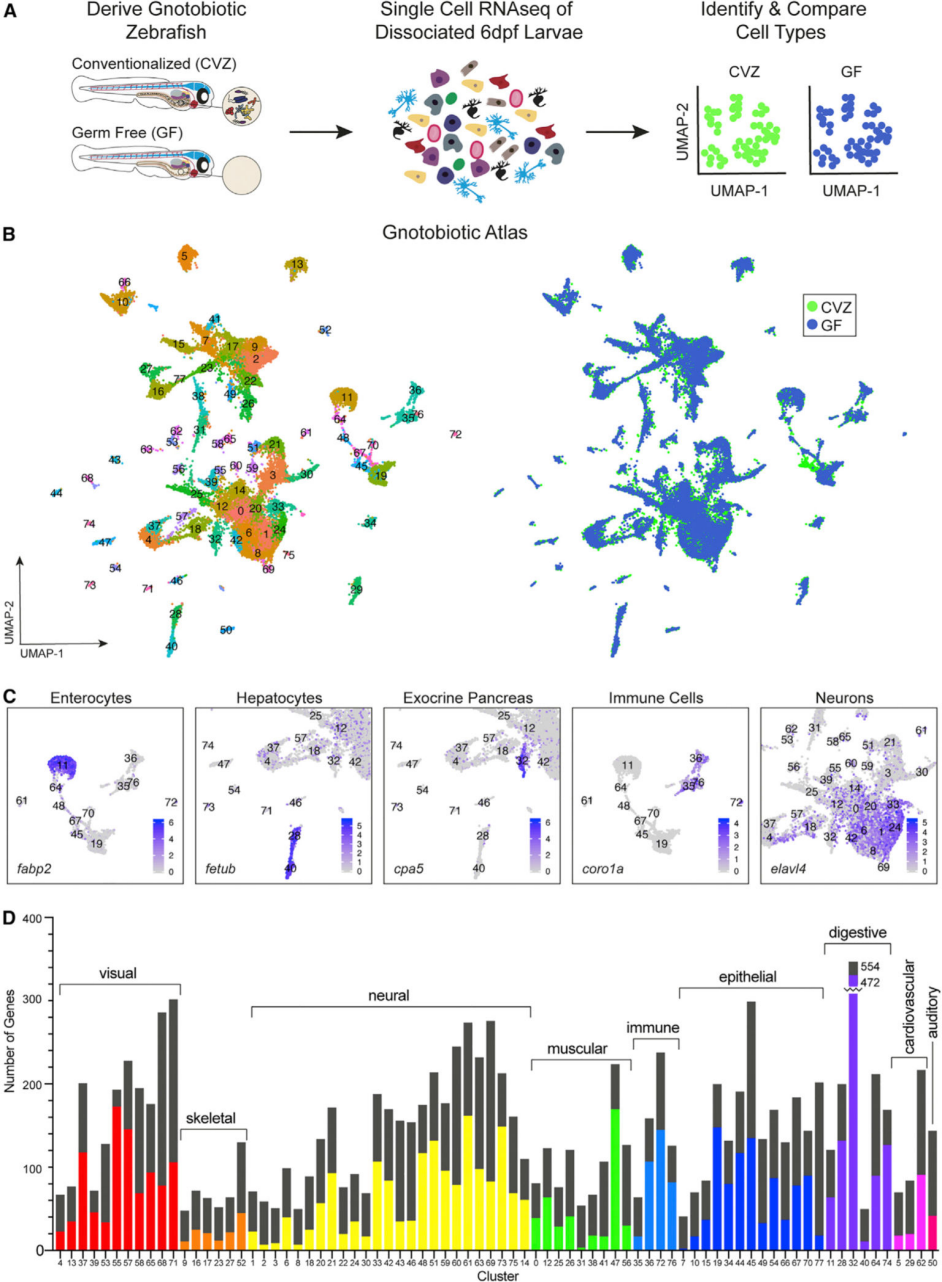
Single-cell transcriptional analysis of whole gnotobiotic larval zebrafish (A) Whole conventionalized (CVZ) and germ-free (GF) 6 dpf zebrafish larvae were dissociated into individual cells prior to single-cell RNA sequencing. (B) Uniform manifold approximation and projection (uMAP) plots display 78 cell type clusters shared between experimental groups. (C) Cell types were identified by their transcriptomic profile; examples (with marker genes) include enterocytes (*fabp2*), hepatocytes (*fetub*), acinar cells of the exocrine pancreas (*cpa5*), immune cells (*coro1a*), and neurons (*elavl4*). (D) Host cell types differentially respond to the presence of the microbiota throughout the body as illustrated by the total number of genes significantly enriched within each experimental group (p < 0.05). The height of the bars represents the total sum of differentially expressed genes in CVZ versus GF cells for each cluster. The colored portion of each bar represents the number of genes significantly expressed within CVZ cells and the remaining black portion of the bars represents the number of genes significantly expressed in GF cells (minus the colored portion).

The small size and tractable gnotobiology of zebrafish larvae afford an unprecedented opportunity to survey the responsiveness of every vertebrate host cell type to the presence of their microbiota. We derived sterile zebrafish embryos and reared them germ-free (GF) or conventionalized (CVZ) them with parental tank water. At 6 days postfertilization (dpf), whole larvae were dissociated into single cells and processed for single-cell RNA sequencing (RNA-seq) (Figure 1A). This analysis yielded 33, 992 cells that averaged 23,485 mean reads/cell, with 879 median genes/cell, 2,396 unique transcript molecules (UMI)/cell, and 22,953 and 22,898 total genes detected from the CVZ and GF groups, respectively (Table S1, 10X_RunSummary).

On the basis of transcriptional profiles, our dataset is composed of diverse host cell types with largely equal representation from the CVZ and GF groups, referred to hereafter as the Gnotobiotic Atlas (Figure 1B; Table S1, IdentitySummary). We used the Seurat package in R to integrate cells from both treatments,^5^ identify common sources of variation between CVZ and GF cells, and perform a linear reduction of the data by principal-component analysis (PCA). In the integration strategy, matching cells from each dataset are anchored together on the basis of the similarity of their transcriptional profile. Within integrated datasets, clusters that include similar numbers of cells from each treatment group represent the pairing of cells in a similar biological state. The JackStrawPlot, ElbowPlot, and HeatMap functions included in Seurat deciphered which principal components (PCs) represent a robust compression of the data (Figures S1 and S2). To confirm clustering of transcriptionally similar cells across experimental groups by the Seurat integration strategy, we aligned 6 dpf CVZ cells with different developmental time points (1, 2, and 5 dpf) of whole-organism dissociations from the Zebrafish Single Cell Atlas, referred to hereafter as the Mapping the Developmental Zebrafish Atlas^4^ (Figures S3A and S3B). As anticipated, cells from our 6 dpf CVZ zebrafish aligned more consistently with 5 dpf cells of the atlas compared with 1 and 2 dpf cells.

By integrating CVZ and GF cells together in the Gnotobiotic Atlas, we generated lists of significantly enriched genes shared by CVZ and GF cells for each cluster (Table S1, ClusterX), allowing identification of different cell types present in both treatment groups (Table S1, IdentitySummary). Cell type identities were assigned on the basis of tissue location annotations from the Zebrafish Information Network (ZFIN)^6^ of the top enriched genes for each cluster and annotations^4^ from the Developmental Zebrafish Single Cell Atlas (Table S1, ClusterX) (Figure 1C). This annotation established that the dataset contains diverse cell types from all major organ systems, enabling us to compare transcriptional differences between CVZ and GF treatments for cells across the vertebrate body.

Comparing differentially represented transcripts between CVZ and GF cells within the Gnotobiotic Atlas for each cluster (Data S1) revealed host cell responses to the microbiota occur across many cell types, not limited to cells in direct contact with microbes (Figure 1D). Additionally, the high variability in the number of enriched genes across cell types demonstrates that different cells respond differently to the microbiota even within a tissue type. One cluster in the Gnotobiotic Atlas, number 45, lacked GF cell representation (Figure 1B) in a pattern that was robust to different permutations of PC number (Figure S4A). This cluster is enriched for genes typically found within epithelia (Table S1, Cluster45), such as *epcam*, and Gene Ontology (GO) analysis showed a signature of tissue regeneration and immune activation (Figure S4C; Data S2), including expression of *anxa1a*, *anxa1c*, *agr1*, and *apodb*. Although some of the cells express markers of skin epithelia, such as different types of *keratins*, the cluster lacks a strong signature of tissue identity (Figures S4B–S4D). On the basis of this gene expression pattern and further analysis of dissected intestines discussed below, this cluster appears to consist of progenitor cells from multiple epithelial tissues that co-cluster on the basis of their common cellular responses to microbiota associated with tissue growth and regeneration programs. The underrepresentation of these cells from germ-free tissues may represent a paucity of progenitor cells or a failure of these cells to co-cluster because of reduced expression of tissue regeneration genes. The absence of this cell population in the GF dataset is consistent with the microbiota’s role in stimulating epithelial cell proliferation in tissues including intestine^7,8^ and skin.^9^ Together, these analyses illustrate that cells across the vertebrate body are responsive to the presence of the microbiota, which promotes tissue development. Below we discuss cell-type-specific responses to the microbiota.

### The microbiota stimulates intestinal epithelial transcriptional signatures of cell differentiation and function

**Figure 2.**
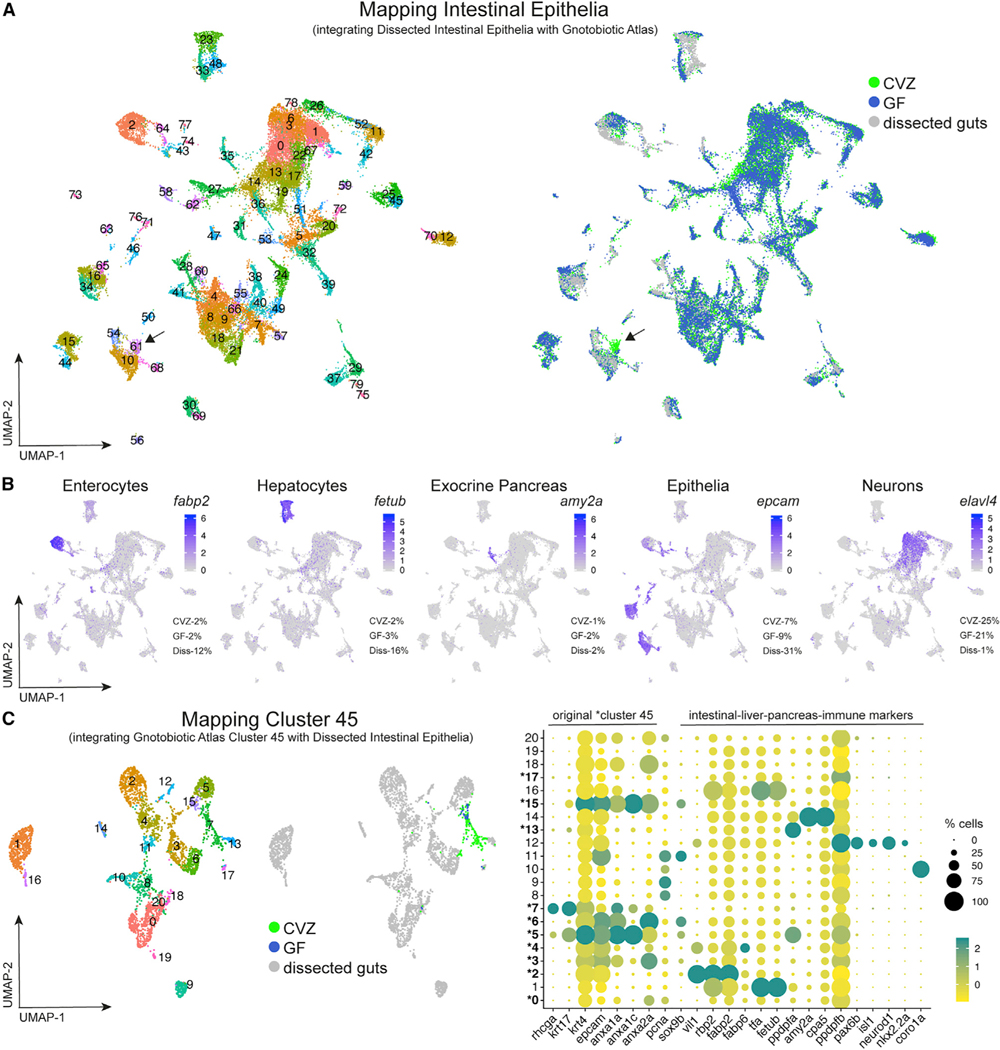
Integration of whole gnotobiotic larval zebrafish cells with dissected larval digestive system cells (A) uMAP plots display integration of single cells dissociated from whole larvae of the Gnotobiotic Atlas and dissected larval guts. (B) Clusters populated by cells from each experimental group confirm and illustrate digestive cell types from dissociation of whole larvae including enterocytes (*fabp2*), hepatocytes (*fetub*), acinar cells of the exocrine pancreas (*amy2a*), and epithelial cells (*epcam*). The percentage of cells within designated clusters, illuminated by different biomarkers, with respect to total cells for each experimental group are shown. Cells from dissected digestive systems primarily populated clusters from whole larvae annotated as digestive system cell types and were a small percentage of the neuron (*elavl4*) clusters, verifying the integration strategy. (C) uMAP plots show that cells from original epithelial cluster 45 include a minority of cells likely to be of the digestive system. The dot plot, and all following dot plots included in the manuscript, displays both the percentage of cells within a cluster or subcluster expressing a transcript (dot size) and expression level of the transcript (dot color). Expression of genes within this dot plot for a subcluster is relative to all other subclusters. Bolded subcluster numbers designated with an astersk within the dot plot indicate that the subclusters include cells originating from cluster 45 within their subcluster population. Dissected Intestinal Epithelia data were derived from GF larvae to mitigate contamination during the length of time needed to dissect a sufficient amount of sample. Arrows point to Cluster 61 in Mapping Intestinal Epithelia.

Transcriptional responses to the microbiota have been best characterized within the intestine (reviewed by Heppert et al.^10^). To supplement our Gnotobiotic Atlas (Figure 1B) and compare it with previous published analyses, we conducted additional experiments in which we dissected GF larval digestive systems, composed of intestines and associated liver and pancreas tissue, dissociated the cells, and performed single-cell sequencing (Figures S5A and S5B). For this experiment, we dissected digestive systems from GF-derived larvae to minimize contamination within the samples during the time needed to accumulate enough tissue. The resulting single-cell transcriptomic data, referred to hereafter as Dissected Intestinal Epithelia (Figure S5B), were used to cross-validate our annotation of digestive tract clusters within the Gnotobiotic Atlas (Figure 1B). Data from Dissected Intestinal Epithelia (Figure S5B) confirmed our annotation of intestinal epithelium, liver, and pancreas cells within our Gnotobiotic Atlas (Figures 2A and 2B). The clusters resulting from integration of Dissected Intestinal Epithelia with the Gnotobiotic Atlas is hereafter referred to as Mapping Intestinal Epithelia. Comparing our lists of enriched transcripts in digestive tract clusters from the Gnotobiotic Atlas (Figure 1B) with previous microarray^8^ and single-cell RNA-seq analysis of dissected CVZ and GF larval zebrafish digestive tracts^11^ revealed good concordance between these datasets (Tables S2 and S3; Figure S6).

### Progenitor cells in the CVZ dataset include digestive system cells

By Mapping Intestinal Epithelia (Figures 2A and 2B), we observed that CVZ epithelial cells originating from Gnotobiotic Atlas cluster 45 maintained their co-segregation into a cluster assigned as 61 but containing fewer cells than the original 45. We hypothesized that cells from the Gnotobiotic Atlas cluster 45 (Figures 1B, S4B, and S4C) had clustered with digestive cell types enriched in the Dissected Intestinal Epithelia dataset (Figure S5B). To investigate this possibility, we computationally isolated Gnotobiotic Atlas cluster 45 cells and used Seurat to integrate them alone with the Dissected Intestinal Epithelia, referred to hereafter as Mapping Cluster 45 (Figure 2C; Data S3). This integration strategy, as was used in Mapping Intestinal Epithelia, will match common cell types between the datasets, to decipher if Gnotobiotic Atlas Cluster 45 is composed of different types of epithelia within Dissected Intestinal Epithelia. A majority of CVZ cells originating from the Gnotobiotic Atlas cluster 45 segregated to cluster 7 of Mapping Cluster 45, indicating that most cells from Gnotobiotic Atlas cluster 45 are extra-intestinal, likely including skin cells. However, 12% of the CVZ cells from Gnotobiotic Atlas cluster 45 spread to diverse digestive system subclusters across Mapping Cluster 45 (Figure 2C), suggesting that Gnotobiotic Atlas cluster 45 includes progenitors of different digestive system tissues.

### Intestinal enterocytes segregate by region and function

**Figure 3.**
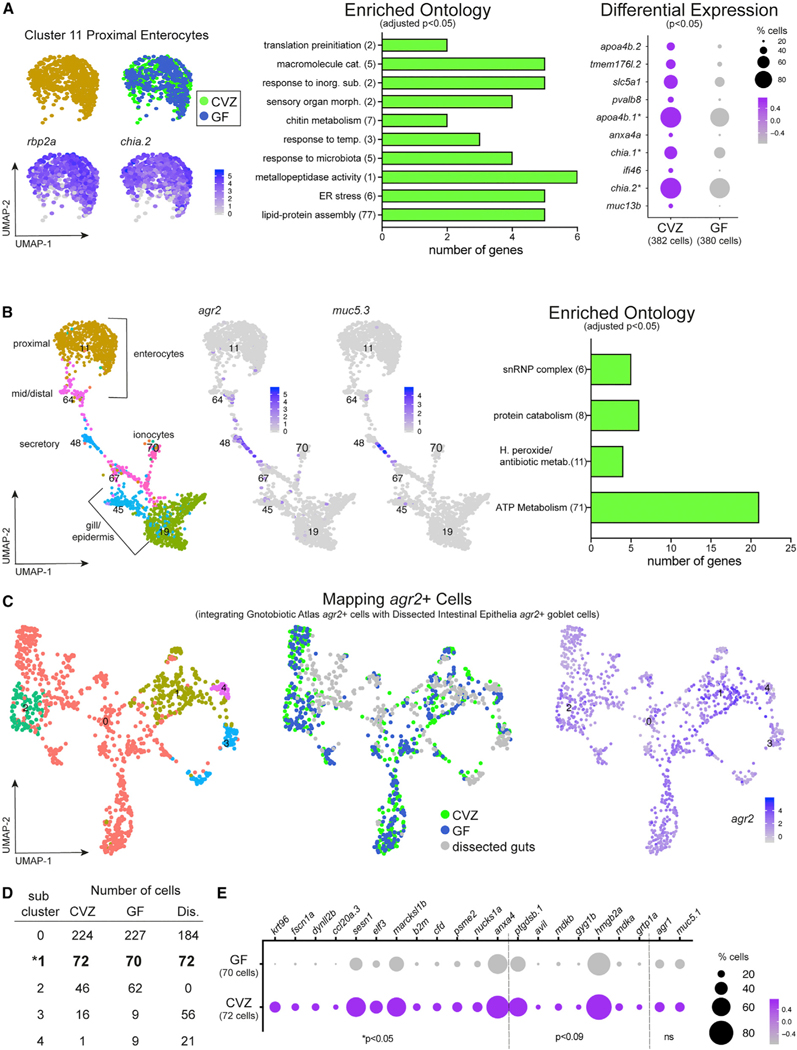
Intestinal enterocytes and secretory cells exhibit cell-type-specific transcriptional responses to the microbiota (A) Cluster 11 is composed of enterocytes from the proximal intestine marked by high expression of *rbp2a* and *chia.2*. GO analysis plot represents gene function categories enriched in CVZ versus GF cells in cluster 11 and the dot plot shows genes significantly enriched within CVZ versus GF cells of cluster 11. Genes included in the dot plot designated with an asterisk correspond to genes included in GO categories. (B) Mucin-secreting cells are localized to cluster 48 indicated by expression of *agr2* and *muc5.3*. GO analysis plot is based on genes significantly enriched within CVZ versus GF cells of cluster 48. (C) uMAP plots display integration of all *agr2*+ cells from whole larvae with *agr2*+ cells from dissected guts. By uMAP and feature plots, subcluster1 displays the most overlap of cells from each experimental group and the highest expression of *agr2*. (D) Subcluster 1 demonstrates consistent pairing of cells from each experimental group from the integration. (E) Dot plot illustrates enriched expression of several genes within CVZ cells of subcluster 1. GO plots in this figure and all following figures use bars to show the total number of genes included in a GO category. The number in parentheses displayed after a GO category title on the y axis signifies the number of GO terms binned into the category. GO terms are considered significant at p < 0.05 after a false discovery rate (FDR) p-corrected adjustment.

Within the Gnotobiotic Atlas (Figure 1B), we identified two adjacent clusters of proximal and distal intestinal enterocytes on the basis of region-specific genes (defined by Lickware et al.^12^) including the proximal intestine genes *rbp2a* and *chia.2* (cluster 11; Figure 3A; Table S1, Cluster11) and the ileum-specific *fabp6* (cluster 64; Figure S7A; Table S1, Cluster64). To further delineate intestinal epithelial cell types within clusters 11 and 64 (Figure S7A), we isolated and re-clustered the cells within clusters 11 and 64 (Figures S7B and S7C; Data S4), which resolved the proximal, mid, and distal intestine described by Lickware et al.^12^ and Wen et al.,^13^ and was consistent with further analysis of the Zebrafish Atlas intestinal epithelia clusters.^14^ For this resource manuscript, we included lists of differentially expressed genes between CVZ and GF cells within these discreet region-specific subclusters of intestinal epithelia and their resulting GO analyses (Data S5). Of the Gnotobiotic Atlas, GO analysis of significantly enriched genes within cluster 11 of CVZ versus GF enterocytes revealed that the microbiota stimulates specific cellular responses (Figure 3A; Data S1, Cluster 11, Clu11_GOsorted_CVZup.xlsx). We observed enrichment of genes involved in macromolecule catabolism, metallopeptidase activity, and lipid-protein assembly, including *apoa4b.1* and *apoa4b.2*, consistent with previous work illustrating that the microbiota regulates fatty acid metabolism and intestinal absorption.^15^ We also saw induction of responses to microbes such as chitinase genes *chia.1* and *chia.2* and other cellular responses consistent with microbial challenge including responses to inorganic compounds, temperature, and endoplasmic reticulum (ER) stress (Figure 3A). Enrichment of *chia.1*, *muc13b*, and *tmem1761.2* within CVZ versus GF enterocytes was also observed in an analysis of differential gene expression between CVZ and GF 6 dpf dissected digestive systems.^14^ Consistent with our annotation of cluster 64 (Figure S7A) as mid intestine enterocytes, which includes lysosome rich enterocytes with high capacity for absorption and processing of luminal proteins,^16^ we observed significant enrichment within CVZ cells of genes involved in coated vesicle and proteosome complex formation (Data S1, Cluster 64, Clu64_GOsorted_CVZup.xlsx).

### Intestinal goblet cells segregate with other mucus-secreting cells

Goblet cells within the intestinal epithelium secrete mucins into the lumen, providing a nutrient source for microbes as well as a mucus barrier between the luminal microbiota and the epithelium. *Agr2* is an endoplasmic reticulum protein disulfide-isomerase that is highly expressed in mucus secretory cells including goblet cells of the zebrafish intestine, pharynx, and epidermis.^17^ Clusters 50 and 48 from the Gnotobiotic Atlas (Figure 1B) showed the highest enrichment for *agr2* (~60% and ~30% *agr2*+ cells, respectively) (Table S1, Cluster50 and Cluster48). To identify intestinal goblet cells within the *agr2*+ cell populations, we looked for expression of zebrafish orthologs of *Muc2* and *Muc5*, mucins specific to mouse intestinal goblet cells.^18^ We did not observe *muc2.2* expression in either the Gnotobiotic Atlas or Dissected Intestinal Epithelia nor within the Developmental Zebrafish Atlas.^4^ However *muc5.1* and *muc5.3* expression were enriched within Gnotobiotic Atlas cluster 48, which lies between the intestinal and epidermal epithelium clusters (Figure 3B; Table S1, Cluster48). GO analysis showed significant enrichment of genes involved in hydrogen peroxide and antibiotic metabolism within CVZ cells of cluster 48 relative to GF (Figure 3B; Data S1, Cluster 48, Clu48_GOsorted_CV-Zup.xlsx), consistent with protective responses elicited in cells in close proximity to microbes. Because expression of *muc5.3* in zebrafish has been found within the esophagus and intestine,^19,20^ we speculate that cluster 48 includes goblet cells from tissues other than the intestine.

To refine our identification of intestinal goblet cells, we analyzed *agr2* expression in the Dissected Intestinal Epithelia dataset (Figure S5). The *agr2*+ population within cluster 4 was enriched for intestinal epithelial markers, suggesting that these are intestinal secretory cells (Figure S5B). Using the Seurat integration method, we combined all CVZ and GF *agr2*+ cells from the Gnotobiotic Atlas (Figure 1B) with the Dissected Intestinal Epithelia cluster 4 (Figure S5B) cell population, hereafter referred to as Mapping *agr2*+ Cells (Figure 3C). The *agr2*+ cells from the Gnotobiotic Atlas only partially integrated with the agr2+ cluster 4 cells from the Dissected Intestinal Epithelia, consistent with *agr2*+ cells composed of mixed secretory cell populations, not solely from intestinal epithelia. In the Mapping *agr2*+ Cells analysis, cluster 1 exhibited the tightest co-clustering of CVZ and GF cells from the Gnotobiotic Atlas cells with the cells of Dissected Intestinal Epithelia, suggesting these CVZ and GF from the Gnotobiotic Atlas are intestinal goblet cells (Figures 3C and 3D). Within Mapping *agr2*+ Cells cluster 1, CVZ cells had a significant enrichment of genes involved in cytoskeleton architecture (*krt96*, *fscn1a*, and *dynll2b*), immune responses (*ccl20a.3*, *sesn1*, and *b2m*), and transcription factor activity and development, including Notch signaling (*elf3*, *marcksl1b*, *cfd*, *psme2*, and *nucks1a*), compared with GF cells (Figure 3E; Data S6). Although not statistically significant, several genes involved in developmental pathway signaling and growth (*ptgdsb.1*, *avil*, *mdkb*, and *mdka*), glycogen synthesis (*gyg1b*), innate immune response (*hmgb2a*), vesicle trafficking (*grtp1a*), and markers of goblet cell function (*agr1* and *muc5.1*) were increased. The increased expression of genes involved in the Notch pathway is consistent with our previous finding that the microbiota promotes goblet cell fates through regulation of Notch signaling.^21,22^

### The microbiota stimulates functional maturation and activation of the immune and neural cells

#### Immune cells

**Figure 4.**
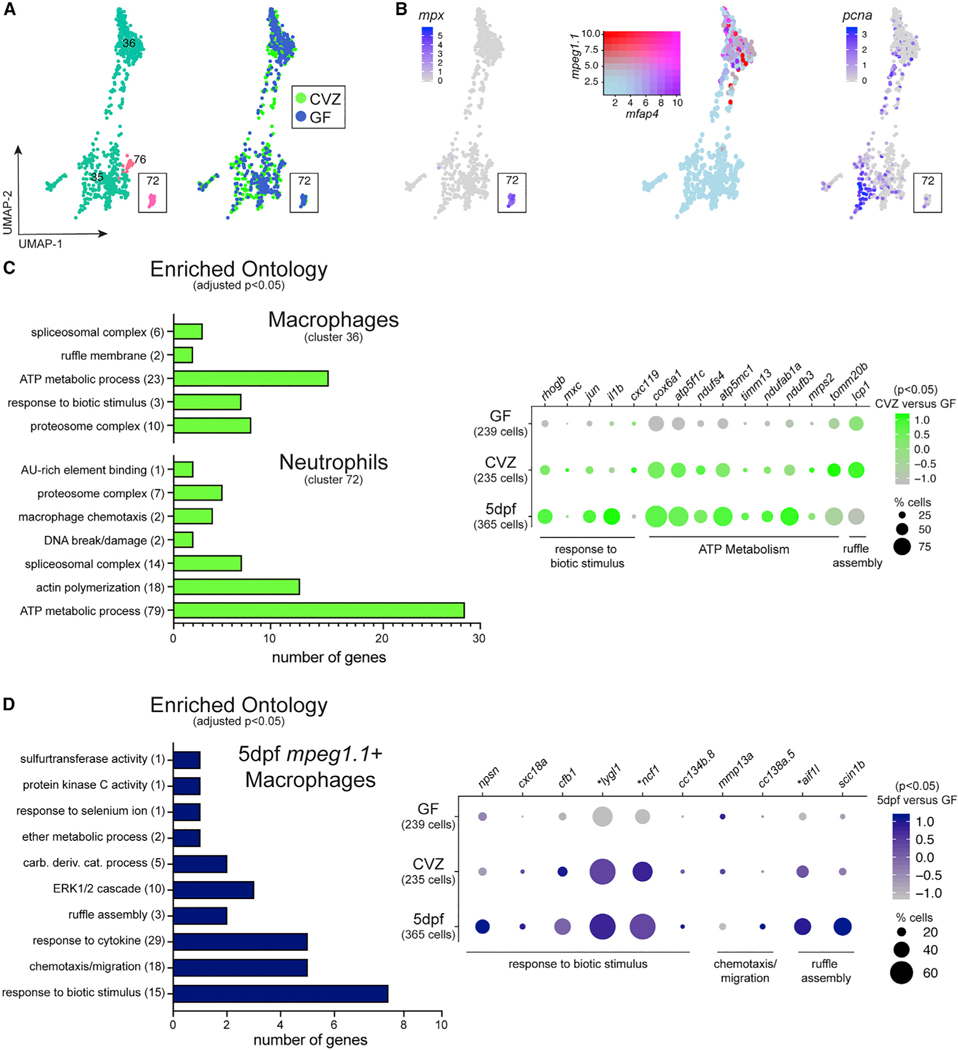
Immune cells exhibit characteristic transcriptional responses to the microbiota (A and B) Clusters 35, 36, 72 and 76 (A) include several immune cell types that differentially express (B) neutrophil biomarker *mpx* and varying degrees of co-expression of macrophage biomarkers *mpeg1.1* and *mfap4*. Expression of *pcna* within cluster 35 indicates a population of immune progenitors. (C) GO analysis plot and dot plot represent genes significantly enriched within CVZ versus GF macrophages and neutrophils, showing similar trends in gene expression compared with conventional 5 dpf larvae of the Zebrafish Atlas. (D) GO analysis plot and dot plot represent genes significantly enriched within CV 5 dpf Zebrafish Atlas macrophages and neutrophils, showing similar trends in gene expression compared with 6 dpf CVZ cells. Genes designated with an asterisk were statistically enriched within CVZ versus GF cells.

Immune cells are dedicated to surveilling and responding to microbial cues and previous studies in gnotobiotic zebrafish have illustrated the responses of these cells to microbiota.^21,23–26^ Neutrophils, one of the major immune cell populations in the larval zebrafish, marked by *mpx* segregated to cluster 72 within the Gnotobiotic Atlas (Figure 1B; Table S1, Cluster72; Figures 4A and 4B). Macrophages, marked by *mpeg1.1*, were found in cluster 36 (Figure 1B; Table S1, Cluster36; Figures 4A and 4B). We additionally annotated cluster 35 as immune cell progenitors within the Gnotobiotic Atlas as it showed an enrichment of several genes involved in larval hematopoietic development including *ikzf1* and genes involved in cell cycle regulation and proliferation, such as the proliferation marker *pcna* (Figure 1B; Table S1, Cluster35; Figures 4A and 4B). Cluster 35 also contains markers of lymphocytes of the adaptive immune system, which start to develop during larval life, including *rag1* and *zap70*.^27^ In the neutrophil cluster 72, genes enriched in the presence of microbiota encoded characteristic immune cell functions including threat sensing, chemotaxis, and cell shape changes, and cellular processing (Figure 4C; Data S1, Cluster 72, Clu72_GOsorted_CVZup.xlsx). These findings were similar for CVZ compared with GF *mpeg1.1*+ macrophages from cluster 36 (Data S1, Cluster 36, Clu36_GOsorted_CVZup.xlsx) and were cross-validated comparing gene expression with the Developmental Zebrafish Atlas immune cells (Figures 4C and 4D; Data S7). Among the CVZ enriched genes within the progenitor immune cell population of cluster 35 were genes involved in DNA recombination and break repair (Data S1, Cluster 35, Clu35_GOsorted_CVZup.xlsx), consistent with the microbiota stimulating lymphocyte maturation.

#### Enteric neurons

Mounting evidence indicates that both the peripheral nervous system (PNS) and CNS branches sense and respond to the microbiota.^28–32^ Our Gnotobiotic Atlas dataset contains many neurons, marked by expression of the panneuronal, postmitotic marker *elavl4* (Figure 1C). Here we focus on intestine-resident neurons of the enteric nervous system (ENS), a major component of the PNS that regulates diverse functions within the intestine including secretion, motility, and homeostasis.^33^

**Figure 5.**
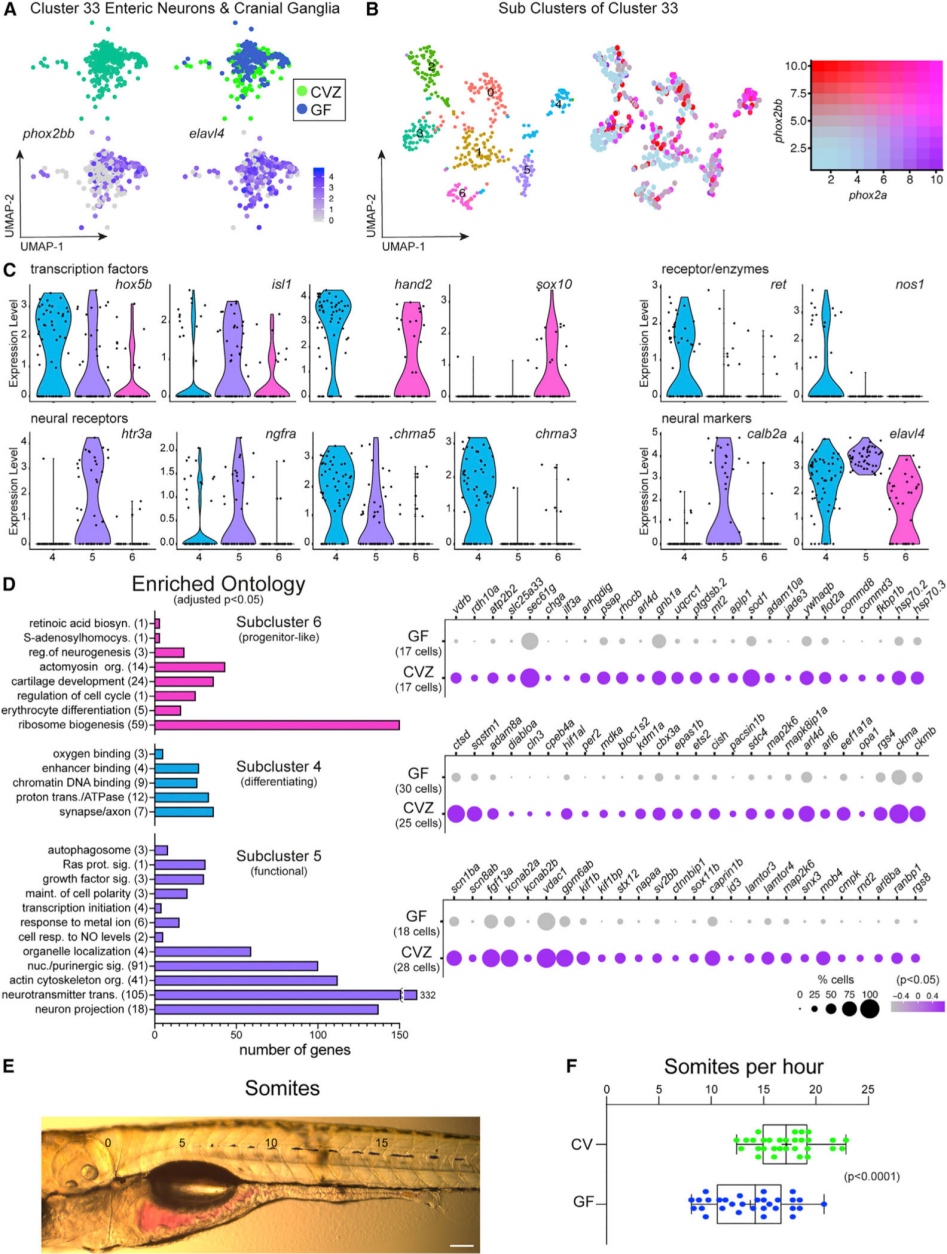
Enteric neurons are transcriptionally heterogeneous in response to the microbiota (A) Cluster 33 includes enteric neurons and cranial ganglia showing expression of *phox2bb* and *elavl4*. (B) uMAP plots show transcriptional heterogeneity of cluster 33 with a range in co-expression of *phox2bb* and *phox2a*. (C) Violin plots compare expression of enteric neuron transcription factors, neural receptors, and neural markers within subclusters 4, 5, and 6. (D) GO analysis plots represent genes enriched within subcluster 4, 5 or 6 relative to the other subclusters. The corresponding dot plots represents differential gene expression in CVZ versus GF cells for each subcluster. (E) Image displays a larval zebrafish post-gavage with phenol red dye. Numbers labeled on the image indicate the somite number. Scale bar of 100 μm corresponds to the micrograph. (F) Horizontal boxplots represent the rate of transit for the red dye post-gavage with respect to somite distance. In the box plots, the middle line representes the median, the right and left sides of the box represent the upper and lower quartiles, the whiskers represent the min and max values, and the plus symbol represents the mean. Each dot symbol represents data from a single 6 dpf zebrafish. The p value displayed is a result from Student’s t test.

On the basis of expression of *phox2bb* and *phox2a*, enteric neurons segregated to cluster 33 of the Gnotobiotic Atlas (Figures 1B and 5A; Table S1, Cluster33). The expression of several other genes characteristic of enteric neurons were also expressed within cluster 33, showing good concordance with previous transcriptional studies of isolated enteric neurons.^34–36^ Cluster 33 is composed of multiple types of PNS neurons as only 52% and 60% of the cells in the cluster express *phox2bb* and *phox2a*, respectively; other top expressed genes in the cluster were identified as biomarkers of cranial ganglia (Table S1, Cluster33). The co-clustering of enteric neurons and cranial ganglia is consistent with both cell types being derived from neural crest. Re-analyzing cluster 33 alone resulted in 7 subclusters, with high co-expression of *phox2bb* and *phox2a* in subclusters 4, 5, and 6 (Figure 5B; Data S8). These subclusters showed expression of transcription factor *hoxb5a*, providing additional evidence they are composed of enteric neurons^37^ (Figure 4C). Transcriptional differences between these three enteric neuron clusters reflected temporal progression of their maturation.^38^ Subcluster 6 is characterized by expression of early ENS fate determinants (*hoxb5a*, *hand2*, and *sox10*) and limited neural receptor and enzyme expression, suggesting this population contains enteric neuron progenitors. In subcluster 4, the combinatorial expression of *elavl4* and *ret*, with little expression of *sox10*, suggests a differentiating population.^38^ Subcluster 4 shares some transcription factor expression with subcluster 6, but increased expression of neural receptors (*ngfra*, *chrna5*, and *chrna3*) and enzymes (*nos1*) is consistent with increasing functionality. Subcluster 5 resembles fully mature enteric neurons lacking expression of *ret* and *sox10*^38^ and enriched in expression of neural receptors (*htr3a*, *ngfra*, and *chrna5*) and neural markers (*calb2a* and *elavl4*).

To further explore identities of these enteric neuron subpopulations, we performed GO analysis on genes significantly expressed within each subcluster of Figure 5B followed by comparing transcriptional differences between CVZ and GF cells for each population (Figure 5D). Within the progenitor-like population of subcluster 6, several genes involved in ribosome biogenesis, regulation of cell cycle and neurogenesis were enriched versus the other subclusters (Figure 5D; Data S9). This cell population expressed several genes involved in defense response to bacteria and viruses, metal ion binding activity and cell-cell adhesion that were enriched within CVZ cells versus GF (Figure 5D; Data S10). Within the differentiating population of subcluster 4, GO analysis revealed an enrichment of genes associated with chromatin DNA binding and the synapse or axon (Figure 5D; Data S11). CVZ cells of subcluster 4 also showed enrichment of genes involved in defense response to microbes, as well as genes involved in cell maintenance and differentiation (Figure 5D; Data S12) GO analysis of genes enriched within subcluster 5 is consistent with mature neurons including: maintenance of cell polarity, purinergic signaling, neurotransmitter transport, and axon projection (Figure 5D; Data S13). The presence of the microbiota induced the enrichment of many genes in CVZ cells including those involved in voltage-gated ion channel activity and neurotransmission (Figure 5D; Data S14). Our analysis of enteric neurons corroborates their transcriptional heterogeneity^38^ and illustrates how cells of different maturation states respond differently to the microbiota.^39,40^ To explore whether the microbiota-induced upregulation of genes associated with neuronal function corresponded to physiological changes in the enteric nervous system, we measured gut motility in CV versus GF larvae using a dye transit assay^40^ (Figure 5E). We found that CV intestines had significantly faster gut transit times versus GF (p < 0.05, Student’s t test), consistent with the microbiota stimulating enteric neuron activity.

#### Central neurons

Our analyses of CNS neurons also showed transcriptional responses to the microbiota that differed between cells in different maturity states. We identified a neuronal population within the Gnotobiotic Atlas likely to be composed of progenitors within cluster 21 (Figure 1B) by expression of the nervous system development gene *ptn* and co-expression of *fapb7a* and *her4.2* (Figures S8A and S8B; Table S1, Cluster21).^4^ Similar to progenitor-like enteric neurons, several genes involved in regulation of neurogenesis were enriched within cluster 21 CVZ neurons, as were genes involved in immune function and development (Figures S8C and S8D; Data S1, Cluster 21, Clu21_GOsorted_CVZup.xlsx). These findings are consistent with mounting evidence implicating connections between immune response and neurodevelopment.^41^ We additionally observed that mature serotonergic and/or dopaminergic neurons in Gnotobiotic Atlas cluster 69 (Table S1, Cluster69) responded to the microbiota by upregulating genes involved in synaptic machinery for dopamine transmission (Figures S8E and S8F; Data S1, Cluster 69, Clu69_GOsorted_CVZup.xlsx). CVZ neurons of cluster 69 also exhibited significant enrichment of genes involved in MAP kinase activity, negative regulation of proliferation, and calcium binding activity, suggesting that the microbiota plays a role in promotes characteristic function of mature neurons (Figure S8G). Together these data suggest the microbiota plays specific and distinct roles within neuronal cell types, depending on their developmental state, and promotes neural functions at all developmental stages, including neurogenesis, neurodifferentiation, and neurotransmission.

#### Host tissues exhibit global patterns of microbiota responsiveness

**Figure 6.**
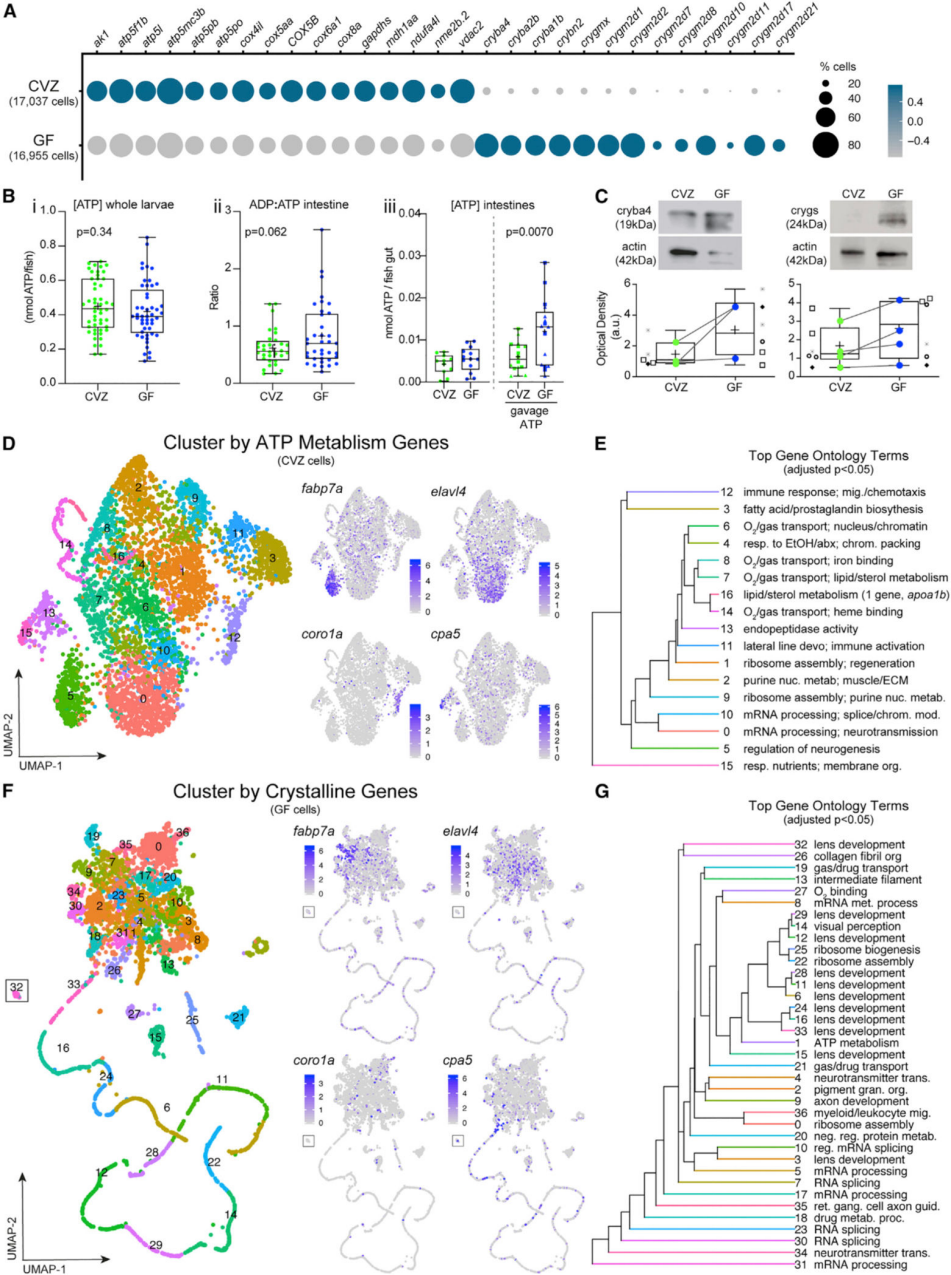
The microbiota induces global patterns of host gene expression (A) Dot plot illustrates global trends of gene expression across all CVZ and GF cells from whole larvae. (B) Boxplots show (i) the concentration of ATP from whole larvae, (ii) the ratio of ADP to ATP of dissected larval intestines, and (iii) the concentration of ATP in dissected CVZ and GF larval intestines alone or 20–40 min following gavage with 1 or 10 mM ATP. Each dot in the measurement of ADP/ATP ratio represents the average to two intestines. (C) Boxplots show the average relative optical density of beta and gamma crystallin protein expression in CVZ and GF larvae. Each symbol represents an individual flask of larvae processed together and matching symbols denote that flasks were from the same gnotobiotic derivation. Colored dots represent average expression between flasks in the same derivation with a line connecting the corresponding derivations between experimental groups. (D) uMAP plots represent CVZ cell clustering on the basis of expression of genes involved in ATP metabolism. Feature plots illustrate cell types maintaining their original cell type identity. (E) Hierarchal tree demonstrates the proximity of uMAP plot cells clustered by expression of ATP metabolism genes and the top GO terms on the basis of enriched genes within a given cluster. (F) uMAP plots represent corresponding GF cells from the original clusters used in (D) clustering on the basis of expression of crystallin genes. Feature plots show that clusters lose their original cell type identity. (G) Hierarchal tree demonstrates proximity of clusters from the uMAP plot of cells clustered by expression of *crystallin* genes and the top GO terms on the basis of enriched genes within a given cluster. Data from *in vivo* experiments, and for the remaining figures, are shown as boxplots with the middle line representing the median, the top and bottom of the box representing the upper and lower quartile, the whiskers representing the min and max values, and the plus symbol representing the mean. Each dot symbol represents data from a single 6 dpf zebrafish, unless otherwise specified. p values displayed on boxplots are from Student’s t test, where p < 0.10 is considered trending and p < 0.05 is considered significant.

Our GO analyses across each of the 78 clusters within the Gnotobiotic Atlas (Figure 1B) revealed two striking patterns. First, ATP metabolism genes such as *atpf51b*, *COX5B*, and *vdac2*, which were widely expressed across all cells, were consistently expressed at higher levels in cells from CVZ animals (Figure 6A). Second, whereas lens-associated *crystallin* (*cry*) genes were almost entirely restricted to the lens cell clusters 39 and 55 in CVZ animals, there was widespread expression of multiple *cry* genes across virtually all GF clusters, predominantly of the beta and gamma type (Figures 6A and S9). We explored each of these transcriptional signatures further.

#### The microbiota elicits cell-type-specific ATP metabolism gene expression patterns

ATP is an ancient molecule whose production spurred one of the earliest examples of host-microbe interactions with the endosymbiosis event that produced mitochondria.^42^ ATP production has been shown to be modulated by the microbiota in many model organisms. In GF fruit flies, whole body ATP levels and mitochondrial respiration are significantly lower than in conventional counterparts.^43^ In mice, ATP levels and mitochondrial respiration are reduced in colonic tissue^44^ and lower in feces from GF animals.^45^ Enriched expression of respiratory electron transport chain genes involved in ATP metabolism was also observed in dissected 6 dpf digestive systems of CVZ versus GF zebrafish larvae.^14^ We measured the concentration of ATP in whole zebrafish larvae and observed extensive inter-individual variation in levels of this rapidly metabolized molecule with no significant difference between GF and CVZ groups (Figure 6B). We explored ATP turnover in dissected larval intestines and found that ADP/ATP ratios varied extensively across individuals, with a trend toward lower ratios in CVZ larvae. We tested the rate of ATP metabolism in the presence and absence of microbiota by microgavaging exogenous hydrolysable ATP into the proximal intestinal lumen, and 20–40 min later dissecting out the intestines and measuring ATP levels. ATP levels were significantly lower in CVZ samples, indicating that ATP was consumed faster within CVZ intestines. These measurements are consistent with the CVZ transcriptional signature of upregulation of enterocyte cellular activities (Figure 3A) and faster transit times of luminal contents (Figure 5F).

ATP production for energy currency or cell signaling can vary extensively across diverse cell types. For example, neurons use massive amounts of ATP for both neural activity^46,47^ and as a signal co-released with various neurotransmitters.^48,49^ Neutrophil production of ATP sustains their immune cell activation and also initiates purinergic signaling for chemotaxis.^50^ Murine colonic macrophages have been shown through single-cell transcriptomic analysis to upregulate oxidative phosphorylation genes in response to the microbiota.^51^ One-third of the clusters in the Gnotobiotic Atlas (Figure 1B), representing diverse cell types, exhibited enriched gene ontologies pertaining to nucleotide and/or ATP metabolism in the CVZ versus GF cells (Figure S10). To better understand how ATP metabolism genes are deployed in response to microbiota, we bioinformatically isolated CVZ cells from the clusters with enriched ATP metabolism genes and re-clustered them using a panel of 228 ATP metabolism-associated genes (Figure 6D), hereafter referred to as Cluster by ATP Metabolism Genes. This analysis revealed 17 new subclusters (Data S15) that largely maintained their original cell type identity, including neural progenitors (84% of original cluster within subcluster 5, *fabp7a*), neurons (73% of original cluster within subcluster 0, *elavl4*), and immune cells (88% of original cluster neutrophils, within subcluster 12, *coro1a*) (Table S4). Some original populations were split into different subclusters, such as acinar cells of the Gnotobiotic Atlas cluster 32 exocrine pancreas with 35% cells within subcluster 15, 19% cells within subcluster 13, and 14% cells within subcluster 0 of the Cluster by APT Metabolism Genes analysis. This suggests heterogeneity of exocrine pancreas ATP metabolism gene use. GO analysis illustrated a diversity of biological processes among the enriched genes within each subcluster of the Cluster by ATP Metabolism Genes analysis (Figure 6E; Data S16), consistent with cell-type-specific functions (e.g., upregulation of chemotaxis in neutrophils). This analysis demonstrates that the microbiota stimulates cell-type-specific ATP metabolism gene expression.

#### The microbiota suppresses cry gene expression across tissues

Crystallins are a heterogeneous group of extremely stable proteins that make up the vertebrate eye lens. In most vertebrates, including zebrafish, rodents, and primates, the lens is composed of alpha crystallin small heat shock proteins and the beta and gamma crystallin superfamily members. The zebrafish genome contains 3 alpha *cry* genes, 13 beta *cry* genes and 40 gamma *cry* genes.^52^ Related *cry* genes were present in early vertebrates prior to lens evolution, implying that they have additional functions.^53^ In both rodents and zebrafish, alpha and beta crystallin are expressed and function in other tissues beside the lens, including the retina, brain, heart, and testes.^53–55^ Within the Tabula Sapiens, several crystalline genes are expressed across diverse human tissue types including epithelia, fast and slow muscle, monocytes, and mucus-secreting cells.^56^ CRYAB is enriched within the large versus small intestine and within human keratinocytes. We confirmed that expression of Cryba4 protein is not restricted to the head or lens of GF larvae but is also detected within the body (Figure S11A). Within whole larvae across several GF derivations, the levels of Cryba4 and Crygs protein were consistently increased in samples of pooled GF larvae (Figure 6C). We note that we did not observe ectopic *cry* transcripts in dissected digestive system cells from GF larvae, nor did Willms et al. (2022)^11^ report an enrichment of *cry* transcript expression in their GF cells from dissected digestive tracts. We suspect that the additional processing of these samples, including incubation of the tissue in enriched media, attenuate *cry* gene expression.

Alpha crystallins function as chaperones that prevent aggregation of client proteins damaged by heator oxidative stress.^55^ Alpha crystallins have antiapoptotic and neuroprotective functions in the retina and other neuronal tissues.^56–58^ In the retinal epithelium, both alpha and beta crystallins stabilize the vacuolar-ATPase, thereby protecting against lysosomal dysfunction.^59,60^ In several mouse models of maternal immune activation, there is a transient upregulation of representative members of all *Cry* gene families within the embryonic brain, consistent with neuroprotection.^61^ In zebrafish, Cryab confers protection against cardiac stress induced by crowding or cortisol.^62^ We explored whether *cry* genes were co-expressed with other chaperone genes in GF cells. Although we saw strong co-expression of *cry* genes with each other, they were not co-expressed with heat shock genes *hsp70.2* and *hsp70.3* (Figure S10B). We also did not observe any relationship between the expression of crystallin genes and ATP metabolism genes (Figure S10B). We next explored how *cry* genes are regulated in the absence of microbiota by re-clustering GF cells of the Gnotobiotic Atlas based solely on the 87 *cry* genes found within the sequencing data, hereafter referred to as Cluster by Crystalline Genes (Figure 6F; Data S17). In contrast to Cluster by ATP Metabolism Genes of CVZ cells, Cluster by Crystalline Genes of GF cells analysis revealed subclusters that largely lost their original cell type identity; for example, neural progenitors (*fabp7a*), postmitotic neurons (*elavl4*), and immune cells (*coro1a*) no longer co-segregated within a specific subcluster (Table S5), showing that *cry* genes are expressed across many cell types in the absence of the microbiota.

Among the GO analysis of Cluster by Crystalline Genes, were reoccurring themes related to cellular architecture (e.g., collagen fibril organization and intermediate filaments) and vesicle trafficking (e.g., neurotransmitter transmission and gas/drug transport) (Figures 6E and 6G; Data S18). Comparing differentially expressed genes across all CVZ and GF cells of the Gnotobiotic Atlas showed that in addition to *cry* genes, several digestive enzymes were enriched within 10–40% of GF cells (Figure S12A). These digestive enzymes include *prss59.1* and *ela2*, which were enriched within 6 dpf GF dissected digestive systems.^11^ We also observed significant co-enrichment of several *keratin* genes and *cry* genes in GF cells (Figures S12A–S12C), which was more pronounced in GF *elavl4*+ cells (predominantly postmitotic neurons) (Figure S12D). Keratins are intermediate filaments that confer structural integrity to postmitotic cells. Intermediate filaments are critical for neural development but their myriad functions have been underappreciated,^63^ such as stabilizing microtubules and inhibiting synaptic vesicle trafficking during synaptic rewiring in postmitotic neurons.^56,64^ We hypothesize that widespread expression of *cry* genes in GF cells counteracts cellular stresses induced by the artificial GF state. Beyond their roles in preventing lens protein aggregation and cataracts, Cry proteins chaperone cytoskeletal components and maintain cytoplasmic organization in diverse postmitotic cells.^65^ In a meta analysis of zebrafish proteins upregulated in response to various biological stressors, Cry proteins are among the top 25 protein families.^66^ More broadly, small heat shock proteins similar to alpha crystallins are abundant in cells experiencing low metabolic states, such as in nematode dauers^67^ and brine shrimp and insects in diapause.^68,69^ Our global transcriptional analysis of GF cells within the Gnotobiotic Atlas suggests that they are less proliferative and less metabolically active than CVZ counterparts, potentially necessitating cellular mechanisms to maintain cytoplasmic organization, prevent protein aggregation, and stabilize stalled, energetically costly processes, such as vesicular trafficking.

#### Exocrine pancreas responses illustrate how the microbiota promotes tissue development and function

**Figure 7.**
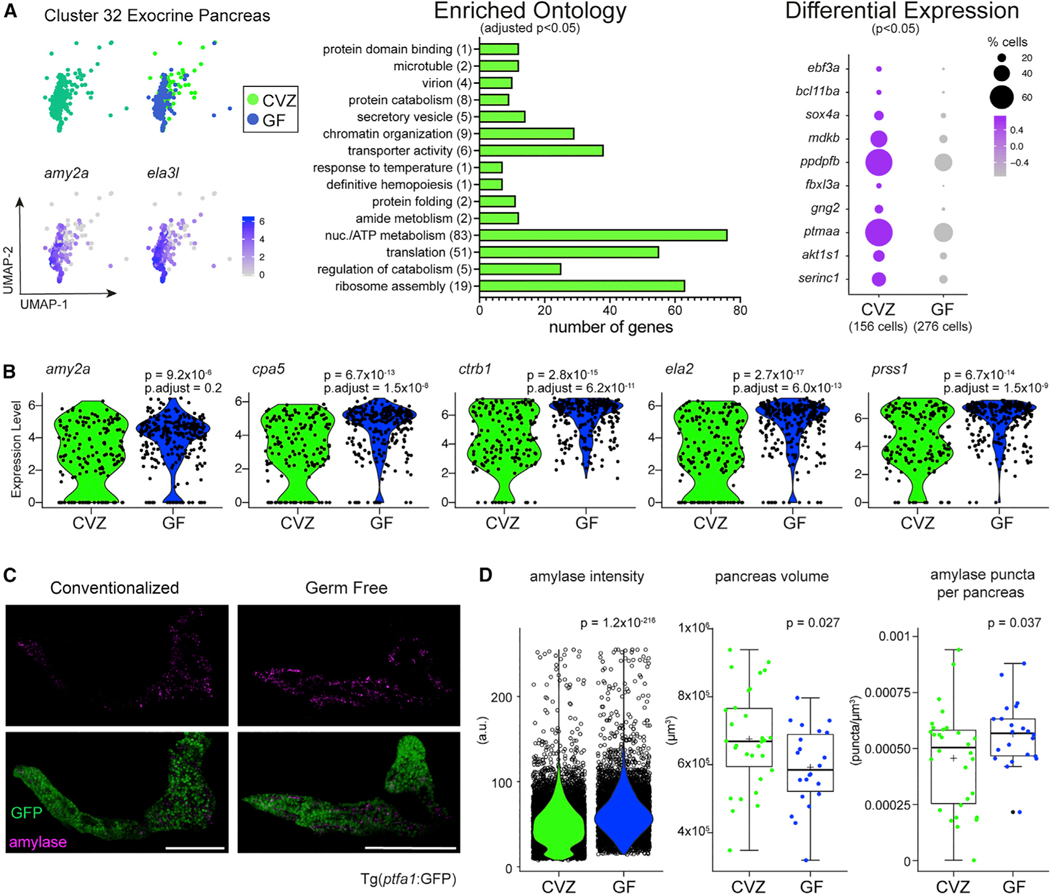
The microbiota promotes tissue development and function within the exocrine pancreas (A) Cluster 32 is composed of acinar cells from the exocrine pancreas showing high expression of digestive enzymes *amy2a* and *ela3l*. GO analysis plot and genes included in the dot plot are based on genes significantly enriched within CVZ cells of cluster 32. (B) Violin plots illustrate the difference in distribution of pancreatic digestive enzyme gene expression between CVZ and GF cells from cluster 32. (C) Images display amylase protein expression within the exocrine pancreas of CVZ and GF larvae. Images are z projections taken by tile-scan with a confocal microscope. For the green channel, intensity levels were increased to illuminate the whole pancreas evenly for publication but had no impact of data generated from the raw images. Scale bar of 100 μm corresponds to the micrographs. (D) The violin plot illustrates the average optical intensity of individual amylase granules and the two box plots show the total volume occupied by the pancreas, and the number of amylase puncta per pancreas between CVZ- and GF-derived 6 dpf larvae. All images were taken with the same optical settings and larvae from each experimental group were imaged on the same day in the same session. Each clear dot in the violin plot represents an amylase+ puncta. Each dot in the subsequent two boxplots for pancreas volume and amylase puncta/pancreas represent individual larvae. p values displayed on plots are from Student’s t tests.

Of all the cell clusters within the Gnotobiotic Atlas, the exocrine pancreas acinar cell cluster 32 (Figures 1B, 1C, and 7A; Table S1, Cluster32), marked by high expression of digestive enzymes including *amy2a* and *cpa5*, had the largest number of microbiota-induced genes (Figure 1D). The exocrine pancreas produces digestive enzymes that are packaged into vesicle-granules and delivered into the intestinal lumen by secretion. GO analysis indicated that the microbiota stimulates a diversity of biological processes in exocrine pancreas acinar cells including secretory vesicle formation, transporter activity, and regulation of catabolism (Figure 7A; Data S1, Cluster 32, Clu32_GOsorted_CVZup.xlsx), consistent with several studies showing associations between exocrine pancreas function and microbiota composition.^70–73^ Pancreatic acinar cells are not only responsible for organ function but also for organ development.^74–76^ Other genes upregulated within CVZ acini included genes involved in DNA-binding transcription factor activity (*ebf3a*, *bcl11ba*, and *sox4a*) as well as growth factor activity and organ development (*mdkb*, *ppdpfb*, *fbxl3a*, *gng2*, *ptmaa*, *akt1s1*, and *serinc1*). Specifically, enriched expression of *ppdpfb* and *mdkb* within CVZ cells suggests that the microbiota promotes pancreas development.

In contrast to the increased expression of genes involved in acinar cell differentiation and function in CVZ cells, we noted a subtle but consistently elevated level of digestive enzyme transcripts in GF acinar cells (Figure 7B). Expression of these digestive enzyme genes was uniformly high in GF acinar cells, whereas CVZ acini shows a bi-modal distribution, consistent with different states of differentiation and maturation. To validate transcriptional increases of digestive enzymes within GF exocrine pancreas, we derived Tg(*ptf1a*:GFP) zebrafish CVZ and GF to mark the exocrine pancreas and stained larvae with an antibody against Amylase to characterize expression of this digestive enzyme (Figures 7C and 7D). The mean intensity of Amylase puncta within the pancreas was lower in CVZ versus GF larvae and displayed a bi-modal distribution similar to the distribution of digestive enzyme gene expression per cell (p < 0.0001, Student’s t test). The average GF pancreas volume was smaller than CVZ (p = 0.027, Student’s t test), with GF pancreases containing more amylase puncta normalized to pancreas volume (p = 0.037, Student’s t test). These analyses reveal how microbiota stimulate both tissue growth and function, promoting developmental plasticity in response to the microbial environment. The transcriptional plasticity of pancreatic cells has also been demonstrated at single-cell resolution within regenerating islets of the zebrafish endocrine pancreas.^77^ In the artificial GF state, the exocrine pancreas appears to be stunted and composed of fully differentiated acinar cells that, on the basis of their transcriptional patterns, are poised in a functionally inactive state, a situation that may require upregulation of cytoprotective Cry proteins to maintain.

## Conclusion

Our analyses reveal widespread and diverse host responses to the microbiota that are not limited to host cells in direct contact with resident microbes. We found that exposure to microbes simultaneously stimulates cell-type-specific programs of microbial responses and metabolic activity, highlighting the close links between immunity and metabolism.^78^ The presence of microbes stimulates cellular programs of proliferation and regeneration in diverse progenitor-like cell types, demonstrating the importance of microbial cues in organismal development. In addition, microbes stimulate the cellular activities of fully differentiated cells from protein-ingesting enterocytes to dopamine-secreting neurons. In contrast, organisms raised in the absence of microbes express unusual repertoires of Cry proteins, suggesting a requirement to stabilize the cytoplasmic organization of fully differentiated but metabolically inactive cells. Collectively our profiling of the cellular transcriptomes of CVZ and GF developing animals reveals myriad roles of resident microbes in stimulating tissue development and cellular functions, demonstrating how microbiota composition and assembly can shape organismal development in a manner that is plastic to the environment the adult organism will inhabit. Our study illustrates the utility of single-cell transcriptomics for exploring intricate interactions between vertebrate hosts and their resident microbes and provides a valuable resource for generating new hypotheses about the impacts of the microbiota on animal development and physiology.

## Limitations of the study

This study surveys the gene expression of all cells of a representative vertebrate in the presence and absence of microbes, but a limitation of this whole body profiling approach is that rare cell types may not be represented in the dataset. Some of the transcriptional responses in the CVZ cells may be specific to the idiosyncratic microbiomes of the animals profiled. Transcriptional responses reported here would require validation using other methods to further explore the underlying biology of the system.

## STAR★METHODS

### RESOURCE AVAILABILITY

#### Lead contact

Additional information and requests for resources or reagents should be directed to the Lead Contact, Karen Guillemin (kguillem@uoregon.edu).

#### Materials availability

This study did not generate unique reagents.

#### Data and code availability

- Single cell RNA sequencing raw FASTQ files have been deposited to the NCBI SRA and are publicly available as of the date of publication with the accession ID PRJNA885906.
- This manuscript does not report original code.
- Any additional information required to reanalyze the data reported is available upon request to the lead contact.

### EXPERIMENTAL MODEL AND SUBJECT DETAILS

#### Zebrafish strains and maintenance

All protocols used for zebrafish experiments were approved by the University of Oregon Institutional Care and Use Committee. Adult zebrafish were maintained using standard husbandry procedures^83^ by the University of Oregon Zebrafish Facility.

Zebrafish embryos were derived germ free (GF) as previously described.^3^ Conventionalized (CVZ) zebrafish were inoculated with parental tank water post GF derivation.

Animals used for the single cell analysis of whole larvae were *Tg*(*nkx2.2a*:EGFP)^79^ and multiple clutches were collected from natural mating of adults from the same tank. The *Tg*(−1.0*ins*:EGFP)^81^ line of zebrafish were used for dissections of the digestive system prior to single cell dissociation. Animals used for western immunoblots were wild-type (ABCxTu strain). The Tg(*ptf1a*:EGFP)^84,85^ line of zebrafish were used for all analyses with the exocrine pancreas. Unless specified, all zebrafish used were 6 days post fertilization (dpf).

### METHOD DETAILS

#### Whole larval zebrafish dissociation

Larval zebrafish were anesthetized with Tricane (Western Chemical, Inc., Ferndale WA). 15 healthy zebrafish with inflated swim bladders were selected for dissociation and put into a sterile 1.5 mL Eppendorf tube. Stock solutions of 10 mg/mL proteinase K (Millipore Sigma, St. Louis, MO) and 10 mg/mL collagenase P (Millipore Sigma, St. Louis, MO) were prepared by dissolving in 1X HBSS (Thermo Fisher Scientific, Waltham, MA). Embryo media was pipetted out from each tube and 37C-warmed 1.3 mL of dissociation solution (0.12 mg/mL proteinase K and 1 mg/mL collagenase P in 1x TrypLE (Thermo Fisher Scientific, Waltham, MA) was added to each tube. Each tube was quickly added to a heat block at 37C, with a timer starter. To dissociate the whole 15 zebrafish per tube into individual cells, each tube was mixed by pipetting 25x every 2 min until ~12 min is on the timer. To stop the dissociation reaction, 200 μl of 4C ‘stop solution’ (5% calf serum, 1 mM CaCl2, and PBS)^4^ was added to each tube, mixed by pipetting 5x, and immediately put into a pre-chilled 4C table-top centrifuge. Dissociated cells were gently spun down for 3 min at 350g and the supernatant removed. Cell pellets were gently rinsed and resuspended with 1mL of 1% BSA in HBSS and pelleted again for 3 min at 350g. The supernatant was removed and the pellet resuspended in 100 μL of 0.04% BSA/HBSS. Cells were then filtered through a pre-chilled 40 μM strainer into a pre-chilled Eppendorf tube, using a pre-chilled 1mL syringe plunger to gently pestle the cells through. An additional 100 μL of fresh 0.04% BSA/HBSS was used to rinse remnants of the strainer into the tube. A Bio Rad TC20 cell counter was used to measure cell concentration and cell viability with Trypan Blue (Bio Rad, Hercules, CA). Additionally, pilot experiments confirmed single cell dissociation with this protocol by visual inspection using the DIC feature of a LEICA DM6 confocal microscope (Leica Microsystems Inc., Buffalo Grove, IL). All groups of dissociated cells had over 80% viability and were diluted to a final concentration of 3,500 cells/ul in 0.04% BSA/HBSS for cDNA library preparation. The timing from euthanasia to handing off dissociated cells for library preparation was under 30 min.

#### Larval zebrafish dissections and dissociation

For larval zebrafish dissections, the entire digestive system (intestine, pancreas, liver) was dissected as previously described.^24^ Briefly, zebrafish were derived germ free and at 5dpf were anesthetized in Tricane (Western Chemical, Inc., Ferndale WA), mounted on a slide and their digestive systems sterilely dissected. Dissected tissue was isolated and put into L-15 culture medium (Thermo Fisher Scientific, Waltham, MA) supplemented with 10% fetal bovine serum (Thermo Fisher Scientific, Waltham, MA), penicillin-streptomycin (MilliPore Sigma, St Louis, MO) and gentamycin (VWR, Radnor, PA). Dissected tissue was incubated overnight in culture media at room temperature prior to single cell dissociations. Approximately 200 larvae were dissected over 3 h.

#### Single-cell cDNA preparation

The University of Oregon Genomics and Cell Characterization Core Facility https://gc3f.uoregon.edu/performed the sample preparation by running the samples on a 10X Chromium Single Cell 3′ platform using v2 chemistry. The goal was to target 10,000 cells per group and the resulting cDNA libraries were amplified with 10 cycles of PCR. The cDNA libraries were first sequenced with experimental groups combined onto a single Hi-seq 4000 lane. This resulted in a low coverage depth. We next had each experimental group sequenced onto its own Hi-seq 4000 lane. The output from all lanes was combined, which reached an optimal number of reads for each sample. All samples were prepared and sequenced on the same days.

#### Western immunoblot

Larval zebrafish were euthanized by tricane and ~30–40 zebrafish were added to an Eppendorf tube. Leftover embryo media was removed from each tube and replaced with 400–600 μL of 4C from a 10mL stock of cold lysis buffer (RIPA buffer (Boston BioProducts, Ashland, MA) and ½ protease cocktail inhibitor (Thomas Scientific, Swedesboro, NJ)). Samples were sonicated with a microtip at 20% amplitude, 1 s pulse on, 0.3 s off for 30 s. This step was repeated until fish were completely dissociated, taking care that a sample would not become warm. Tubes were then put in the freezer for 15 min, followed by thawing on ice prior centrifugation at 14,000 rpm for 20 min at 4C. The supernatant from each tube was isolated and protein concentration for each sample was quantified using the Pierce BCA Protein Assay Kit (Thermo Scientific, Rockford, IL). 20 μg of each sample was loaded onto a 4–20% Gel (Bio Rad, Hercules, CA) for electrophoresis and transferred to a PVDF membrane (GE Healthcare Amersham, Chicago, IL). For cryba4 (Thermo Fisher Scientific, Waltham, MA) detection, membranes were blocked in 5% milk in standard tris base, saline tween (TBST) and then probed with a rabbit polyclonal antibody (Thermo Fisher Scientific, Waltham, MA) at 1:100 in blocking buffer overnight at 4C. For crygs (Thermo Fisher Scientific, Waltham, MA) detection, separate membranes were blocked in 5% BSA in TBST and then probed with a rabbit polyclonal antibody (Thermo Fisher Scientific, Waltham, MA) at 1:100 in blocking buffer overnight at 4C. The resulting bands were visualized with a secondary antibody Anti-rabbit IgG HRP-linked antibody (Thermo Fisher Scientific, Waltham, MA) at 1:1000 for 1 h at room temperature. Membranes where then stripped and re-probed for the loading control actin or tubulin. An Anti-actin polyclonal antibody (Millipore Sigma, St. Louis, MO) made in mouse was diluted at 1:1000 in TBST and incubated with the membrane overnight at 4C. The resulting bands were visualized with a secondary antibody Anti-mouse IgG HRP-linked antibody (Cell Signaling Technology, Dancers, MA) at 1:1000 for 1 h at room temperature. Protein densities for cryba and cryg2 bands were measured and normalized to the protein densities of the corresponding actin bands. Each sample was at least triplicated and averaged according to each gnotobiotic derivation.

#### ATP measurement and gavage

For measuring total ATP concentration of whole larvae, 6/7dpf zebrafish were euthanized by tricane. Individual zebrafish were put into an Eppendorf tube, pipetting out as much excess liquid as possible and 100 μL of 95C distilled water was added to each fish. The tubes were immediately but on the 95C heat-block. All tubes of zebrafish were incubated at 95C for 20–30 min, mixing by pipette every 5 min. All sample carcass remnants were pellet by centrifugation at 14,000 rpm for 30 min at 4C. The ATP concentration of the supernatant was calculated by the ATP Determination Kit (Thermo Scientific, Rockford, IL) relative to a standard. The same protocol was used to measure the ATP concentration of dissected digestive systems of larvae but dissected tissue was boiled in 30 μL of 95C water and centrifugation was omitted. The ADP/ATP Ratio Assay Kit (Bioluminescent) (Abcam, Cambridge, MA) was used to measure the average ratio of ADP to ATP of larvae within dissected digestive systems from 2 zebrafish per sample using the same tissue preparation with 95C water as described above.

To measure ATP turnover in the larval intestine, 6dpf zebrafish were gavaged with 1 or 10mM of ATP (Millipore Sigma, St. Louis, MO), dissolved in embryo media, directly into the proximal intestine as previously described.^86^ Approximately 20–40 min post gavage, the digestive systems of zebrafish were dissected and processed for measuring ATP concentration as described above.

#### Gut transit analysis

Gnotobiotic larvae underwent gavage, as described above, with 4.6 nL of 0.2% phenol red dye solution and gut transit was assessed as previously described.^40^ Briefly, using a dissecting microscope for visualization, the number of somites that the dye has moved was divided by the fraction of the hour that passed. Rate of dye transit was complete once dye was expelled through the distal orifice.

#### Pancreas image analysis

Gnotobiotic larvae Tg(*ptfa1*:GFP) were fixed, stained, and imaged by confocal microscopy as described in Hill et al.^87^ An Anti-Amylase CLONAL antibody (Abcam, Boston, MA, USA) made in rabbit was diluted (1:1000). Images of whole pancreases are composed of z stack through the entire depth of the pancreas using boundaries the *ptfa1* reporter. Larvae from each treatment group were imaged on the same day with the same settings. The volume (*μ*m^3^) of each pancreas and characterization of Amylase puncta were determined using IMARIS version 9.9.0 (Bitplane, South Windsor, CT, USA). For pancreas volume estimation, GFP-positive cells from Tg(*ptf1a*:GFP) CVZ and GF zebrafish were utilized to create a surface estimation of the pancreas with a surface detail of 1 *μ* m and a local Gaussian filter background subtraction with a radius of 30 *μ* m. Amylase puncta were identified with an antibody against Amylase. Similar to pancreas surface creation, Amylase puncta fluorescent signal was utilized to create a surface estimation of each puncta with a surface detail of 1 *μ* m and local background subtraction with a radius of 5 *μ* m. All data were exported from IMARIS as.xls files and processed into final data frames using R version 4.1.3.

### QUANTIFICATION AND STATISTICAL ANALYSIS

#### Computational analysis

The sequencing data were aligned to the zebrafish genome (GFCz11_91) using the 10X Cell-ranger pipeline (3.0.2). The Seurat software package for R (3.1.4) was used to subject the data to standard pre-processing workflow prior to integrating CVZ and GF cells together. The data were filtered such that any cells expressing more than 3,000 genes, including more than 50,000 read counts, or more than 20% mitochondrial genes were not included in the final analysis. The expression level of each gene was normalized by total expression via log-transformation with a 10,000 scale factor. We performed a linear dimensional reduction of the data by principle coordinate analysis (PCA) and calculated 150 principle components (PC). Based on Jack Straw methods to determine significance of PCs, the first 122 PCs significantly explain the variance of the data (p < 0.0001) but by Elbow Plot most of the variation can be explained between 30 and 60 PCs (Figure S1). To better grasp how adding additional PCs impact our data, we performed multiple clustering analyses comparing the inclusion of 30, 60, 90, and 122 PCs (Figure S2) using all genes in the dataset. The data we are reporting in subsequent figures includes 60 PCs with a resolution of 3.0, which produces 78 clusters where we can conservatively identify distinct cell types and avoid possible technical noise.

Gene expression conserved between the gnotobiotic treatments for all the clusters was performed using the FindConservedMarkers function in Seurat. The lists of genes that are conserved between CV and GF treatments and significantly contribute to the segregation of cells into each cluster is made available in the workbook, Table S1. We then isolated the data from specific cell types by sub-setting a cluster(s) or subset cells globally that were positive for a biomarker(s) of interest. Re-analyzing specific cell type populations allowed us to understand the extent of gene expression heterogeneity within a cell population and the FindMarkers function was used to find differentially expressed genes between CV and GF cells in the subpopulations by Wilcoxon rank sum tests. These analyses took into account the expression of all present transcripts within the cells prior to clustering except for the analysis of *elavl4*+ cells which used the top variable genes per the standard workflow in Seurat. Gene ontology (GO) analysis on the lists of differentially expressed genes (p< 0.05) was done using the ClusterProfiler software package for R (3.14.3) and the genome wide annotation for zebrafish org.Dr.eg.db (3.10.0). In the GO analyses, input genes are assigned a GO term and are bioinformatically associated with GO annotations, which provides a statement about the function or biological context of genes based on current biological knowledge http://geneontology.org/. Resulting GO terms were subject to FDR p value correction to limit potential false-positives. To distill the repetitive GO annotations and terms, we manually triaged through each analysis included in the figures and binned similar annotations that were described by the same sets of genes into broader categories. Within figures, GO analyses are shown as bar graphs displaying the number of cumulative genes that contribute to the GO category. All GO terms, genes that contribute to the terms and which terms were binned into our broad categories is made available as supplemental spreadsheets.

#### *In vivo* analysis

Data from *in vivo* experiments are displayed as boxplots with the data median as the line within the box, the top and bottom of the box representing the upper and lower quartile, the whiskers representing the min and max values, and the ‘+’ symbol representing the mean. A Students’ Ttest was used to gauge statistical differences of means between CVZ and GF groups in non-sequencing experiments. For all statistical tests, p < 0.05 is considered significant.

#### Accession of data

The sequences used for analysis in this study were deposited to the NCBI SRA: PRJNA885906. All gene lists and GO term analysis are included in supplemental tables/files.

## Supplementary Material

MMC1

MMC10

MMC11

MMC12

MMC13

MMC14

MMC15

MMC16

MMC17

MMC18

MMC19

MMC2

MMC20

MMC121

MMC22

MMC23

MMC3

MMC4

MMC5

MMC6

MMC7

MMC8

MMC9

MMC24

## Figures and Tables

**Table:** KEY RESOURCES TABLE

REAGENT or RESOURCE	SOURCE	IDENTIFIER
Antibodies		

Rabbit polyclonal anti-pancreatic alpha amylase	Abcam	Cat# ab21156: RRID: AB_446061
Rabbit polyclonal anti-CRYBA4	Thermo Fisher Scientific	Cat# PA522032: RRID: AB_11154203
Rabbit polyclonal anti-CRYGS	Thermo Fisher Scientific	Cat# PA569836: RRID: AB_2689858
Mouse monoclonal anti-beta-Actin	Sigma-Aldrich	Cat# A5316-100UL
Rabbit polyclonal anti-beta Tubulin	Abcam	Cat# ab6046: RRID: AB_2210370
goat anti-rabbit IgG (H + L) secondary antibody Alexa Fluor 488	Thermo Fisher Scientific	Cat# A21450: RRID: AB_2534117
Donkey anti-mouse IgG (H + L) secondary antibody Alexa Fluor 647	Thermo Fisher Scientific	Cat# A-31571: RRID: AB_162542
goat anti-mouse IgG (H + L) secondary antibody Alexa Fluor 488	Thermo Fisher Scientific	Cat# A11029: RRID: AB_2534088
goat anti-rabbit IgG (H + L) secondary antibody, HRP	Thermo Fisher Scientific	Cat# G-21234: RRID: AB_2536530
Horse anti-mouse IgG secondary antibody, HRP	Cell Signaling Technology	Cat# 7076

Chemicals, peptides, and recombinant proteins		

proteinase K	MilliPore Sigma	Cat# 1245680500
collagenase P	MilliPore Sigma	Cat# 11213857001
TrypLE	Thermo Fisher Scientific	Cat# A1217701

Critical commercial assays		

ATP Determination Kit, 200-1000 assays	Thermo Fisher Scientific	Cat# A22066
ADP/ATP Ratio Assay Kit (Bioluminescent)	Abcam	Cat# ab65313

Deposited data		

Raw FASTQ files	This study	NCBI SRA: PRJNA885906

Experimental models: Organisms/strains		

Zebrafish Tg(nkx2.2a:mEGFP) (Pauls et al., 2007)^79^	ZFIN	ZDB-TGCONSTRCT-070117-51
Zebrafish Tg(*ptf1a:eGFP*) (Thisse et al., 2004)^80^	ZFIN	ZDB-GENO-080111-1
Zebrafish *Tg*(−1.0*insulin*:eGFP), (DiIorio et al., 2002)^81^	ZFIN	ZDB-FISH-150901-7130 ZDB-FISH-150901-16611
Zebrafish/Wild-type ABC x Tu strain	U Oregon	N/A

Software and algorithms		

Cell Ranger 3.0.2	10X Genomics	N/A
Seurat 3.1.4	Butler et al., 2018^82^	https://satijalab.org/seurat/
ClusterProfiler 3.14.3	Bioconductor	https://bioconductor.org/packages/release/bioc/html/clusterProfiler.html
org.Dr.eg.db 3.10.0	Bioconductor	http://bioconductor.org/packages/release/data/annotation/html/org.Dr.eg.db.html
R 3.6.0 & 4.1.3	R	N/A
RStudio 1.2.5001	RStudio	N/A
GraphPad Prism 8.2.1	GraphPad Prism	N/A
IMARIS 9.90	IMARIS	N/A

## Highlights

- The Larval Zebrafish Gnotobiotic Atlas: a resource of microbiota-responsive genes
- The microbiota induces transcriptional responses across all tissues of the body
- The microbiota stimulates widespread transcriptional signatures of ATP production
- The microbiota promotes exocrine pancreas growth and functional plasticity
