# Supplementary material for: Cell-type-specific responses to the microbiota across all tissues of the larval zebrafish
Source: Cell Rep. Author manuscript; Available in PMC 2023 Oct 23. (PMC10423310; doi:10.1016/j.celrep.2023.112095)
Supplement: MMC1 [file NIHMS1880944-supplement-MMC1.pdf]

Cell Reports, Volume *42*

Supplemental information

Cell-type-specific responses to the microbiota across all tissues of the larval zebrafish

Michelle S. Massaquoi, Garth L. Kong, Daisy Chilin-Fuentes, Julia S. Ngo, Patrick F. Horve, Ellie Melancon, M. Kristina Hamilton, Judith S. Eisen, and Karen Guillemin

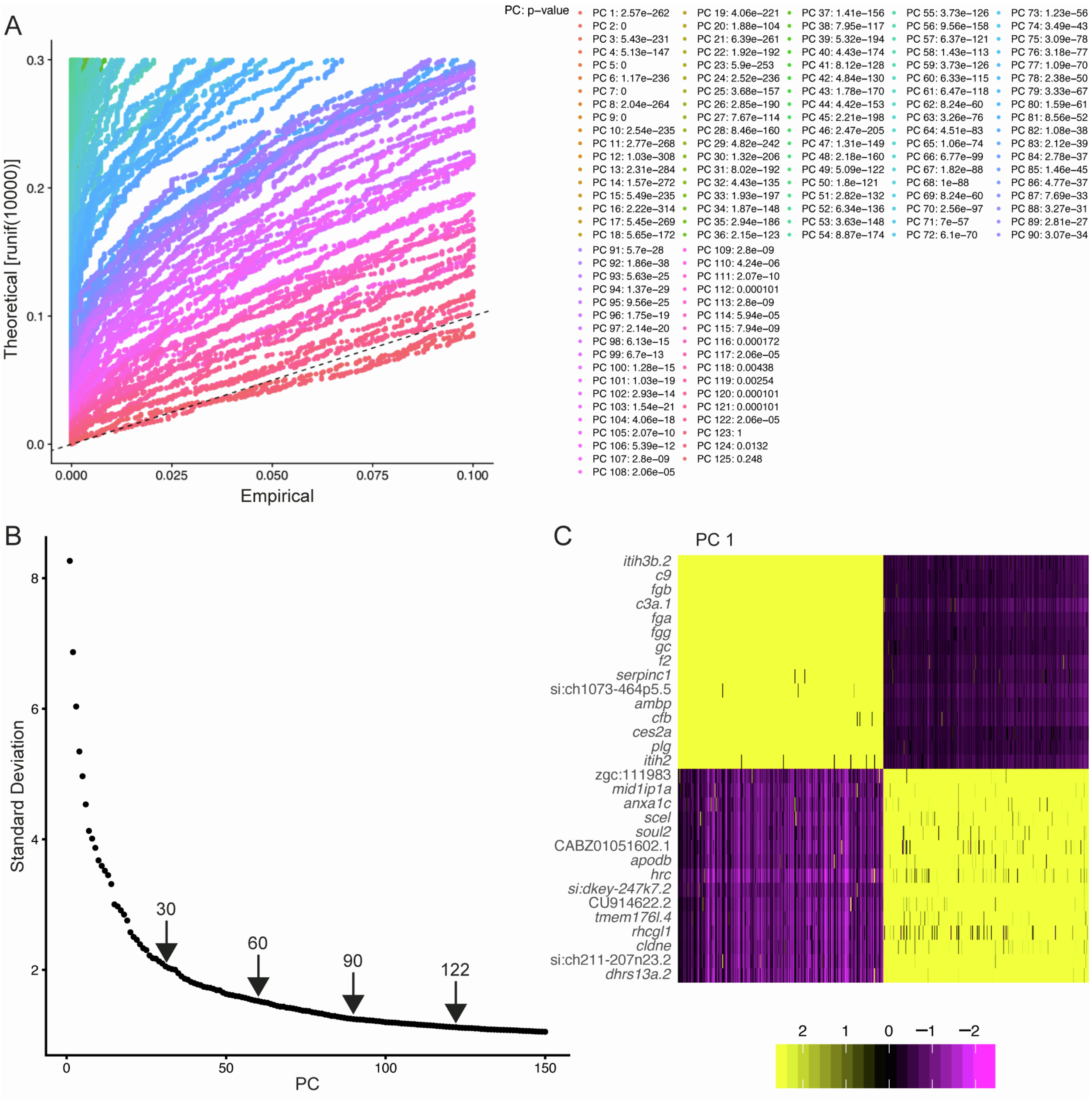

Supplemental Figure 1. Determination of Principle Components for Downstream Analyses, Related to Figure 1. A) Jackstraw plot illustrates the p-value for each principal component (PC) and B) Elbow plot displays how much variation is attributed to each subsequent PC. C) Heatmap of genes significantly contributing to variation within PC1 are those expressed within the liver and integument.

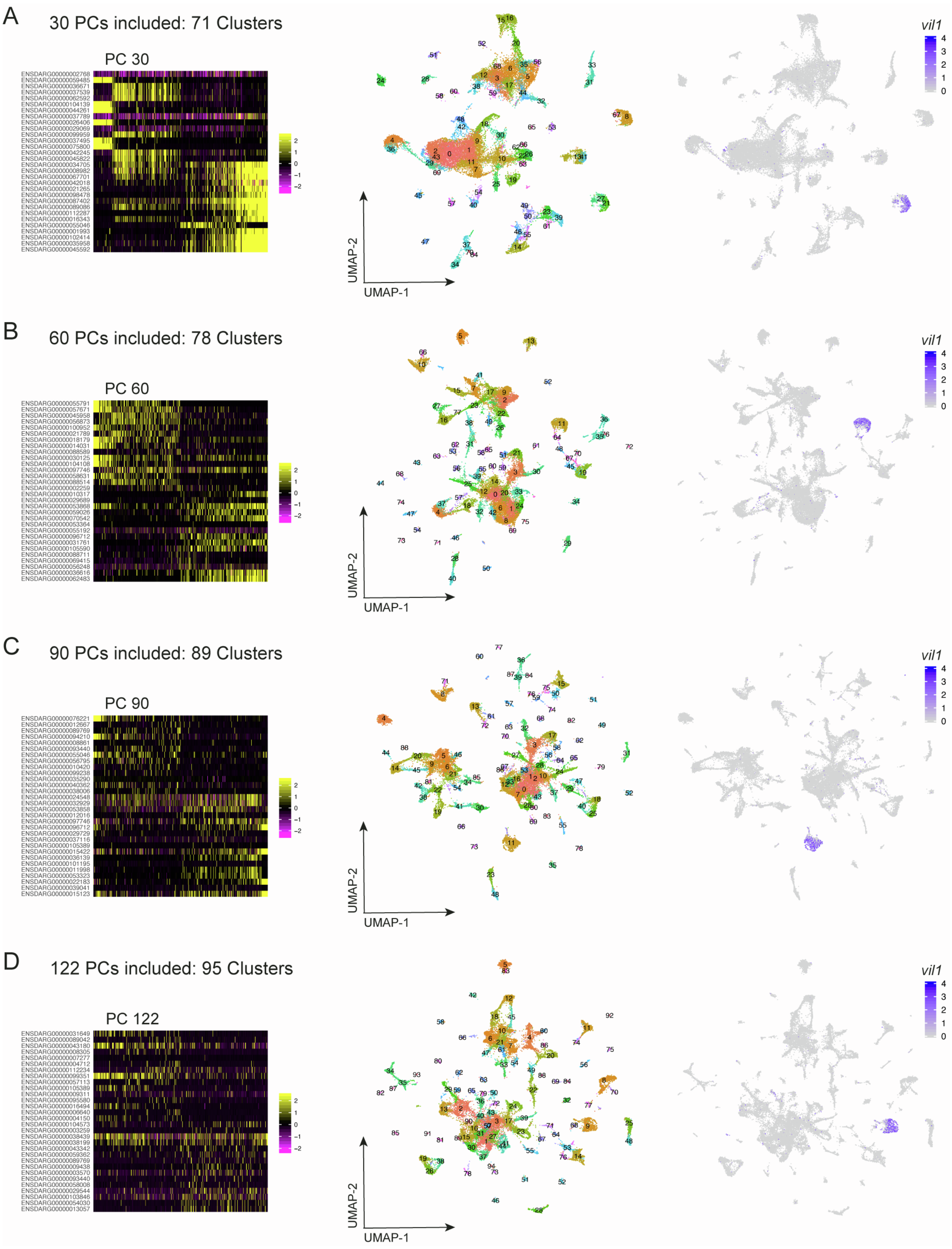

Supplemental Figure 2. Inclusion of Additional PCs Increases the Number of Clusters, Related to Figure 1. Heatmap, resulting uMAP, and expression of intestinal biomarker *vil1* are displayed after inclusion of the first A) 30, B) 60, C) 90, and D) 122 PCs.

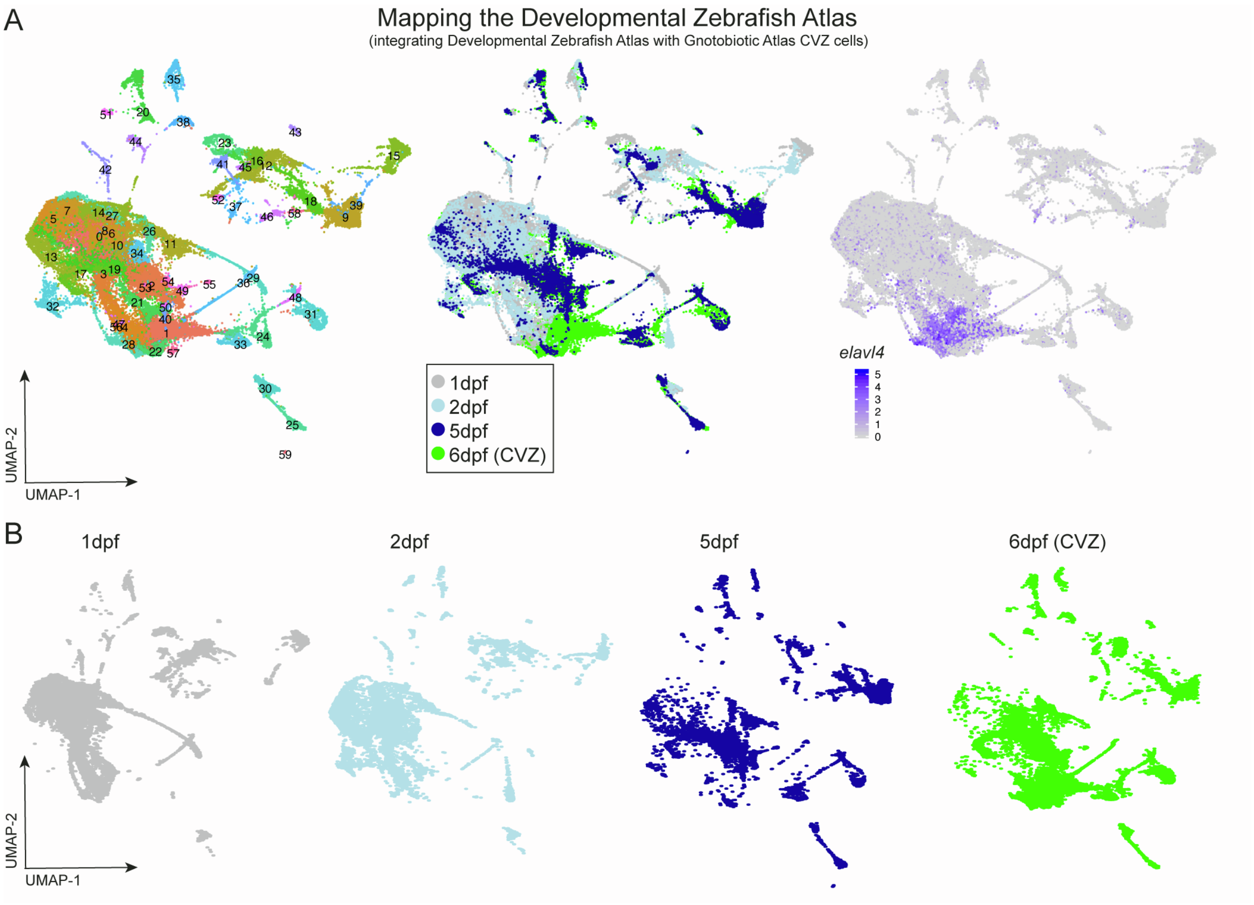

Supplemental Figure 3. Transcriptomic profile of 6dpf CVZ Cells Are Similar to 5dpf Larvae of the Zebrafish Atlas, Related to Figure 1. A) Integration of the Zebrafish Atlas 1, 2, 5dpf whole organism single cell RNAseq data, Farnsworth et al., 2019[4], with CV 6dpf data illustrates that 6dpf cells align more closely with the 5dpf cells than earlier timepoints, with the exception of neural cells (*elavl4*+) which are B) largely absent within the 5dpf data sets, possibly due to differences in cell dissociation protocols.

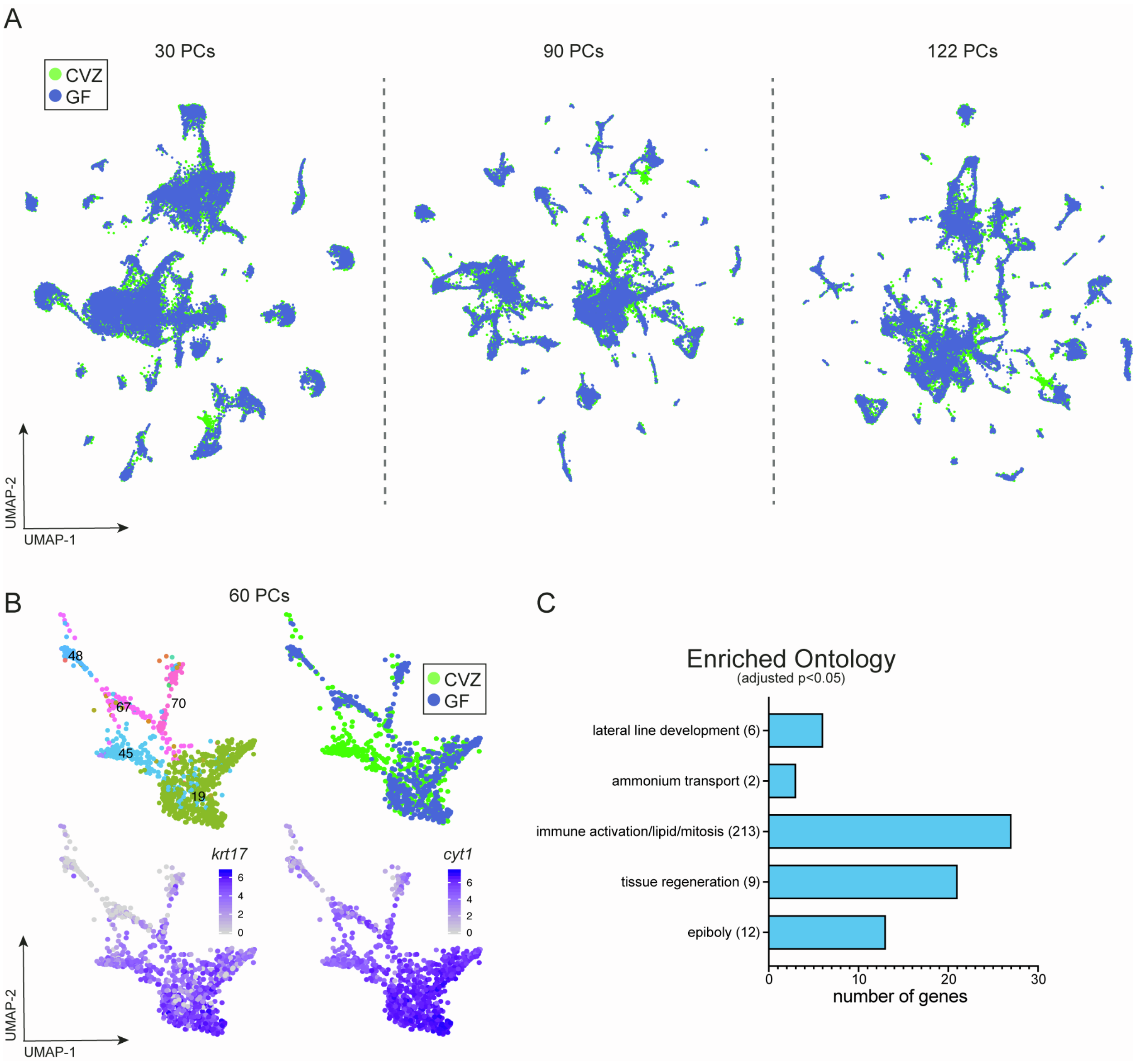

Supplemental Figure 4. GF Zebrafish Lack Representation of an Epithelia Cell Type, Related to Figure 1 & 2. A) uMAP plots display a transcriptionally distinct type of epithelium is overrepresented within CVZ versus GF cells despite different permutations of PC inclusion. B) Cluster 45 has high expression of *krt17* and *cyt1* similar to cluster 19 epithelia. C) GO analysis plot is based on genes enriched within cluster 45 versus all other clusters.

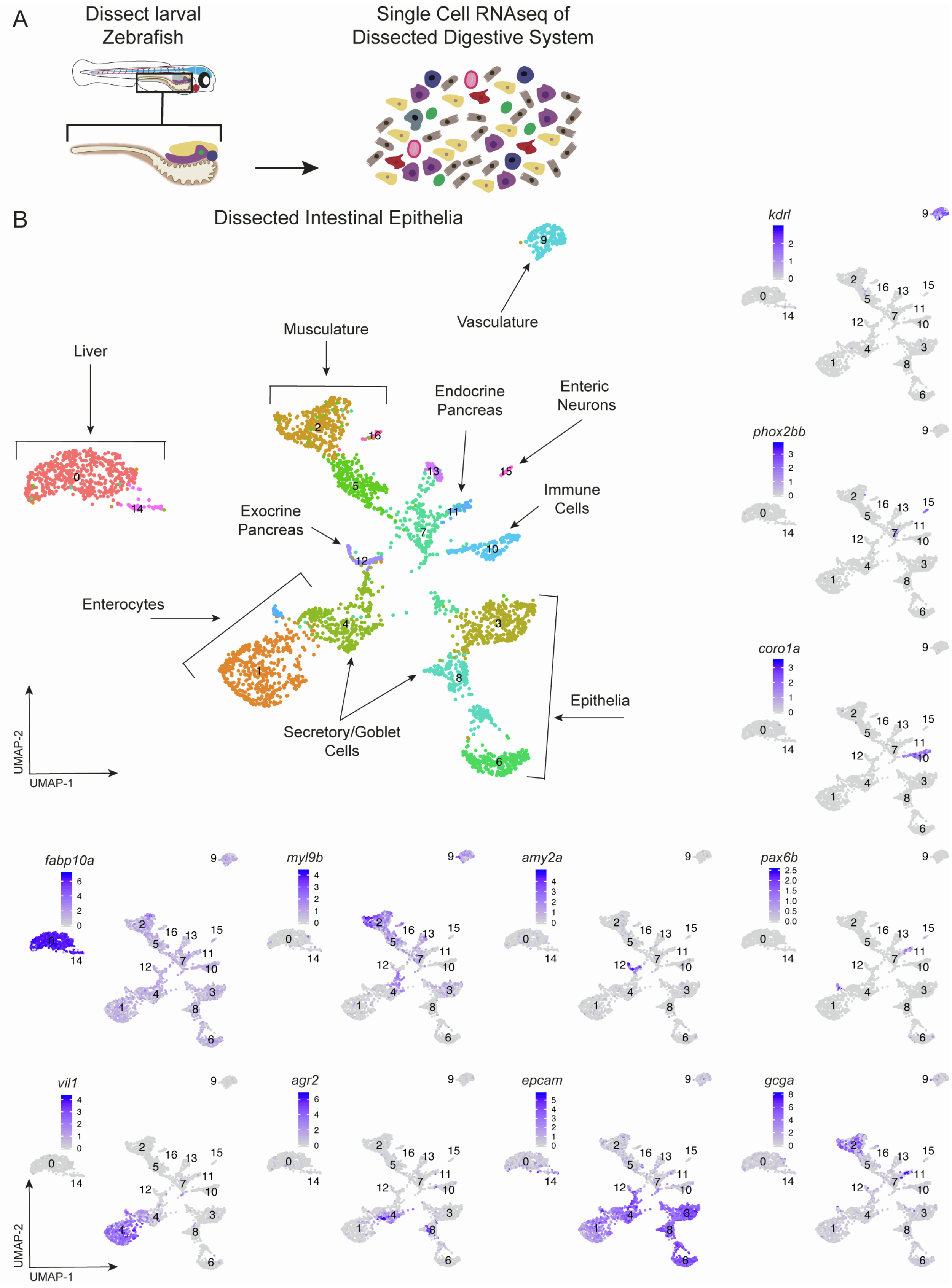

Supplemental Figure 5. Dissected Intestines of Larval Sebrafish Contain a Diversity of Digestive System Cell Types, Related to Figure 2. A) Digestive systems of larval zebrafish were dissected, cells were dissociated and subjected to single cell RNA sequencing. B) uMAPs show diversity and enrichment of enterocytes and associated digestive system cells. Dissected

Intestinal Epithelia data were derived from GF larvae to mitigate contamination during the length of time needed to dissect a sufficient amount of sample.

Comparing Enriched Gene Expression in CV Cells

(Rawls et al., 2004 Microarray on Dissected Guts versus Single Cell Clusters)

Similar Trend

Opposite Trend

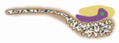

Rawls et al 2004 DEGs	Cluster 11 & 64 Enterocytes	Cluster 28 & 40 Liver	Cluster 48 & 61 Secretory Cells	Cluster 33 Enteric Neurons	Cluster 32 Exocrine Pancreas	Cluster 72 Neutrophils	Cluster 36 & 76 Macrophages
ANXA2	anxa4	anxa4	anxa1a	anxa1a	anxa1a	anxa4	anxa1a, anxa11b
AP1S1					ap2m1a		ap1s2, ap2s1, ap3s2
Apob	apoa4b.1, apoa4b.2 (2x), apoc2,	apoc1, apom	apoa1b, apoa2	apoa1b, apoa2, apoe	apoc1, apoda.2	apoa1b, apoa2, apoc1, apom	
	apoa1b, apoa2						
ARP2	arpc1a				arpc2		
C3		c3a.3					
C4		c4b					
Calr[g]	calr		calr3a	calr	calr		
CBX1		cbx7a			cbx1a, cbx1b, cbx7a		
CORO1C						coro1a	
Dlc2		dynll2b					
DNAJB11		dnajc5aa	dnajc5aa	dnaja2a, dnajc7, dnajc8	dnaja2b, dnajc5aa		
			dnajb12a				
Gpx2	gpx1b	gpx1a	gpx1a, gpx1b				gpx1a
			gpx4a				
HMGA1	hmgb1b	hmgb1a, hmgb1b	hmga1a, hmgb1b		hmgb1a, hmgb1b		
HMGN2		hmgn7			hmgn2, hmgn6, hmgn7		
HSPD1[h]	hsp10.1, hsp70.2, hsp90b1, hspa5	hsp70.2, hsp90aa1.2	hsp90b1	hsp70l	hsp70l, hsp90ab1	hsp90ab1	hsp70.2, hsp70.3
HSPD1[h]		hspd1	hspd1				
IF2	eif2s1b, eif3ha	eif1b, eif4a1b, eif4bb					
	eif3c, eif3d, eif3ja, eif3m, eif4abp2						
Ifit1	ifi45, ifi46		ifi46				
KPNA2			kpna3				
LSM6			lsm5, lsm6				
MAPRE1[g]	map1ab				map1aa, map1lc3b, map4l	mapre1a	
Mcm5			mcm5				
MFAP4							MFAP4 (1 of many)
MSN	msna						
NUCKS		nucks1a			nucks1a		
PABPC1			pabpc1b		pabpc1a		
Pcna			pcna				
PFDN2		pfdn1			pfdn1	pfdn6	
PHB	phb						
PPP1R3B			ppp1r7		ppp2cb, ppp2r1ba		ppp2cb
PPP4R2	ppp3r1b						
Psma5	psma1, psma3, psma6a		psma4, psma8		psma3, psma4, psma5	psma3	psma4, psma5, psma6a, psma6l
Psmb3			psmb4	psmb1, psmb6		psmb1	psmb6
Psmd12	psmd2, psmd14	psmd6	psmc6		psmd1, psmd3	psmd7, psmd11a	
Psme3	psme2		psme1				psme1, psme2
PTGDS	ptgdsb.1						
SDF2L1				sdf2l1			
SF3B4		sf3b6	sf3a2				sf3a1, sfb1
SMARCA5			smarce1		smarce1		
SNRPD1			snrpd1		snrpb, snrpd2		snrpd1, snrpd2
			snrpd2				
SNRPE						snrpe	
SPC18	spcs3						
SRI						sri	
TOMM34			tomm20b	tomm5			tomm20b
Tpm3	tpm1				tpma		
TSPAN-1	tspan13a, tspan15	tspan3a			tspan2a, tspan3a, tspan7b		
UBE2N	ube2na	ube2e3	ube2ib	ube2ia	ube2ib, ube2v1	ube2ia	ube2v1
ZNF259		znf395b	ZNF276	znf536	znf395a, znf395b, znf593, znf609b		

Supplemental Figure 6. Comparison of Differentially Expressed Genes in Gnotobiotic Microarray Versus Single Cell RNA-sequencing of Digestive Systems, Related to Figure 1 and Table S2. Common genes between dissected gnotobiotic intestines of 6dpf zebrafish larvae

from Rawls et al., 2004[8] microarray analysis and cell types from single cell dissociations likely included in dissected digestive systems. Congruent genes highlighted in green indicate a similar enrichment within the respective CVZ treatments whereas genes highlighted in red indicate the opposite trend, increased expression within GF single cells. Comprehensive gene expression comparisons between gnotobiotic groups across Rawls et al., 2004[8] and Willms et al., 2022[11] dissected digestive systems scRNAseq data is included in Table S2.

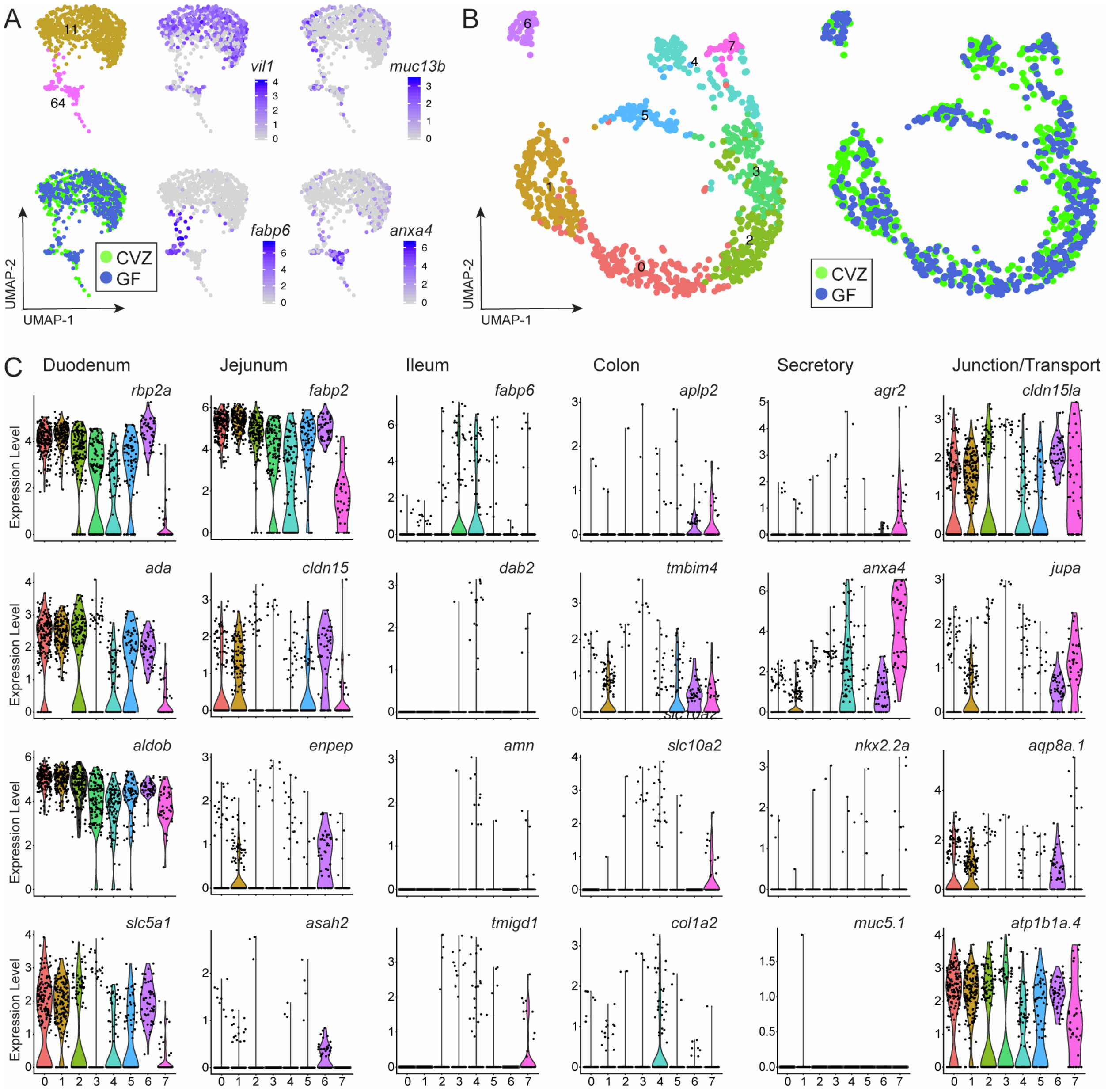

Supplemental Figure 7. Enterocytes Transcriptionally Segregate by Proximal to Distal Localization Along the Intestine, Related to Figure 3. A) Cluster 11 and 64 are composed of intestinal enterocytes with enriched expression of *vi11*, *muc13b*, *fabp6* and *anxa4*. B) uMAP plots show transcriptional heterogeneity of intestinal enterocytes from cluster 11 and 64 which C) segregate by expression of proximal to distal intestinal epithelial biomarkers.

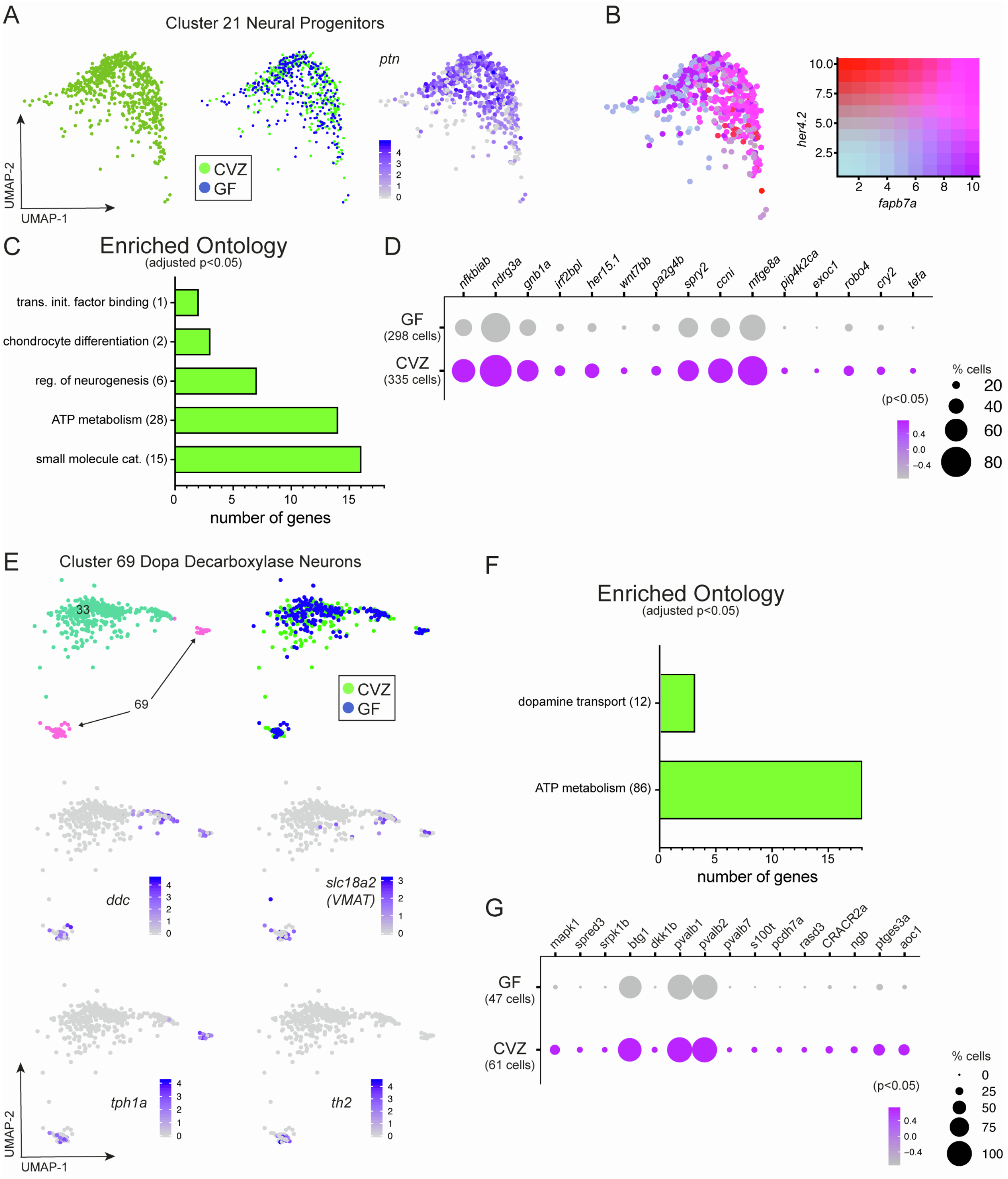

Supplemental Figure 8. CNS Neurons Respond to the Microbiota, Related to Figure 1. A) Cluster 21 is composed of neural progenitors showing enriched expression of progenitor marker *ptn* and B) co-expression of *fapb7a* and *her4.2*. C) GO analysis plot and D) dotplot based on enrichment of genes within CVZ versus GF cells of cluster 21. E) Cluster 69 is populated with serotonergic and dopaminergic neurons displaying expression of *ddc*, *slc18a2*, *tph1a,* and *th2*. F) GO analysis plot and G) dotplot based on the enrichment of genes within CVZ versus GF cells of cluster 69.

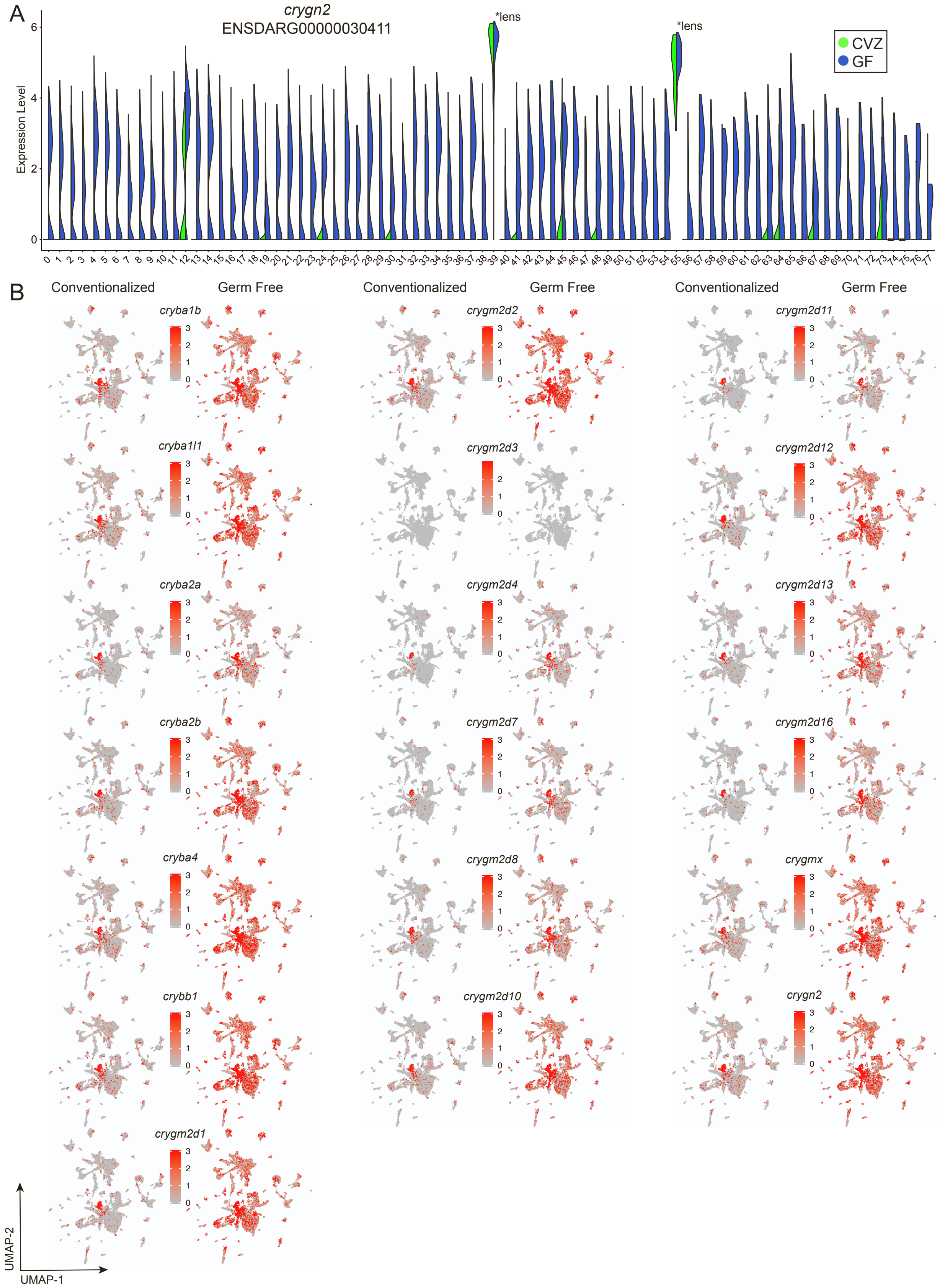

Supplemental Figure 9. Crystallin Genes are Enriched within GF Cells, Related to Figure 6. A) Violin plot shows expression of *crygn2* within lens fiber clusters 39 and 55 with enriched expression in GF cells across all clusters. B) uMAP plots compare global expression of *crystallin* in CVZ and GF cells.

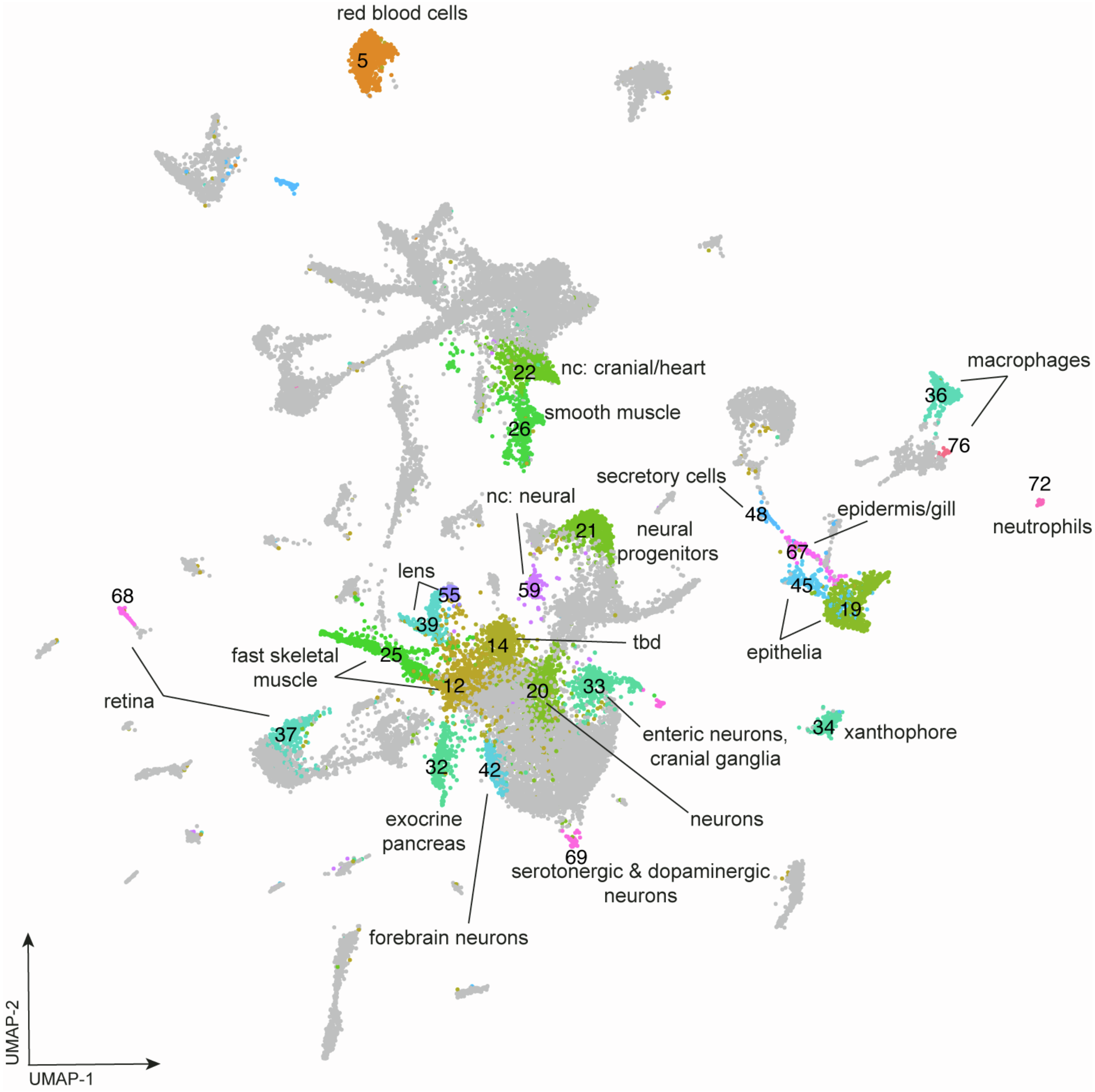

Supplemental Figure 10. The Microbiota Promotes Expression of Genes Involved in ATP and Nucleotide Metabolism in Diverse Cell Types, Related to Figure 6. uMAP highlights clusters composed of diverse cell types that show enrichment of gene expression involved in ATP metabolism within CVZ versus GF cells.

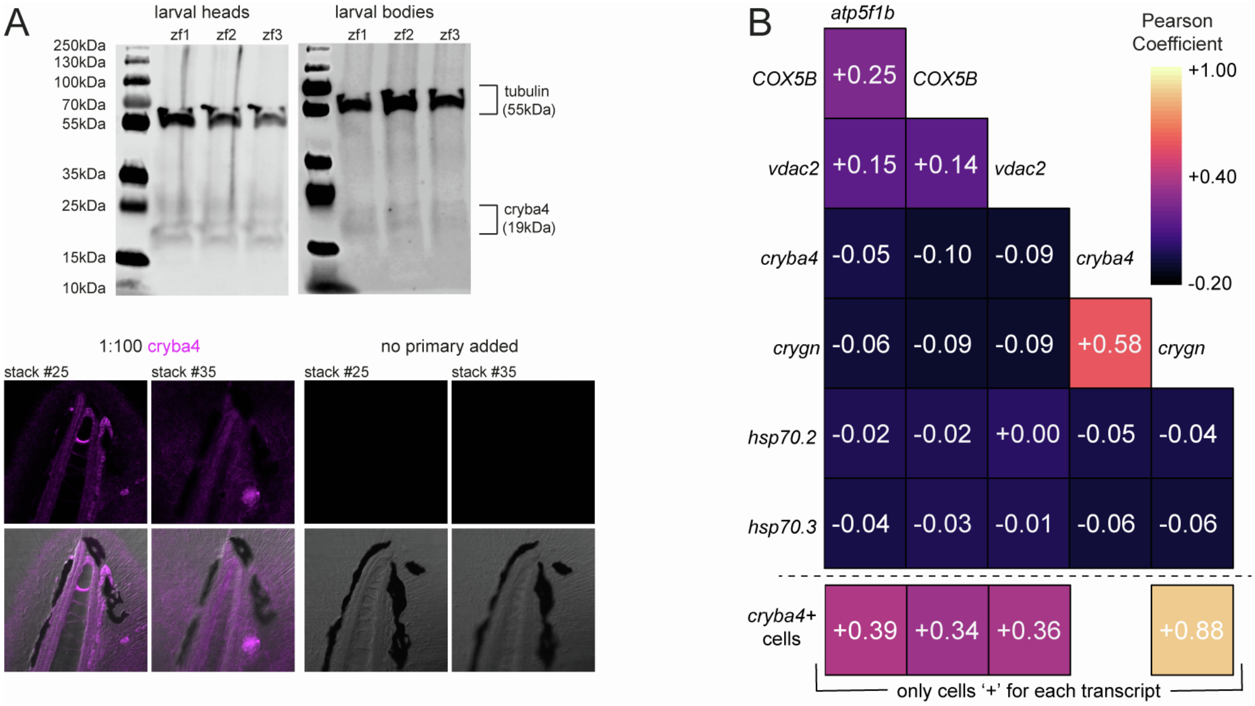

Supplemental Figure 11. Crystallin Expression Occurs Outside of the Lens in GF Larvae and is Uncorrelated with Heat-shock and ATP Metabolism Genes, Related to Figure 6. A) Western blot showing protein expression of Cryba4 within heads versus corresponding bodies of GF larvae. Images display expression of Cryba4 in different tissue structures of the larval tail. B) Heatmap plot showing Pearson Correlational coefficients across all CVZ and GF cells in the experiment with respect to *cryba4* and *crygn* to heat-shock and ATP metabolism genes. Heatmap also displays Pearson Correlational coefficients in analyses that only include cells positive for both transcripts.

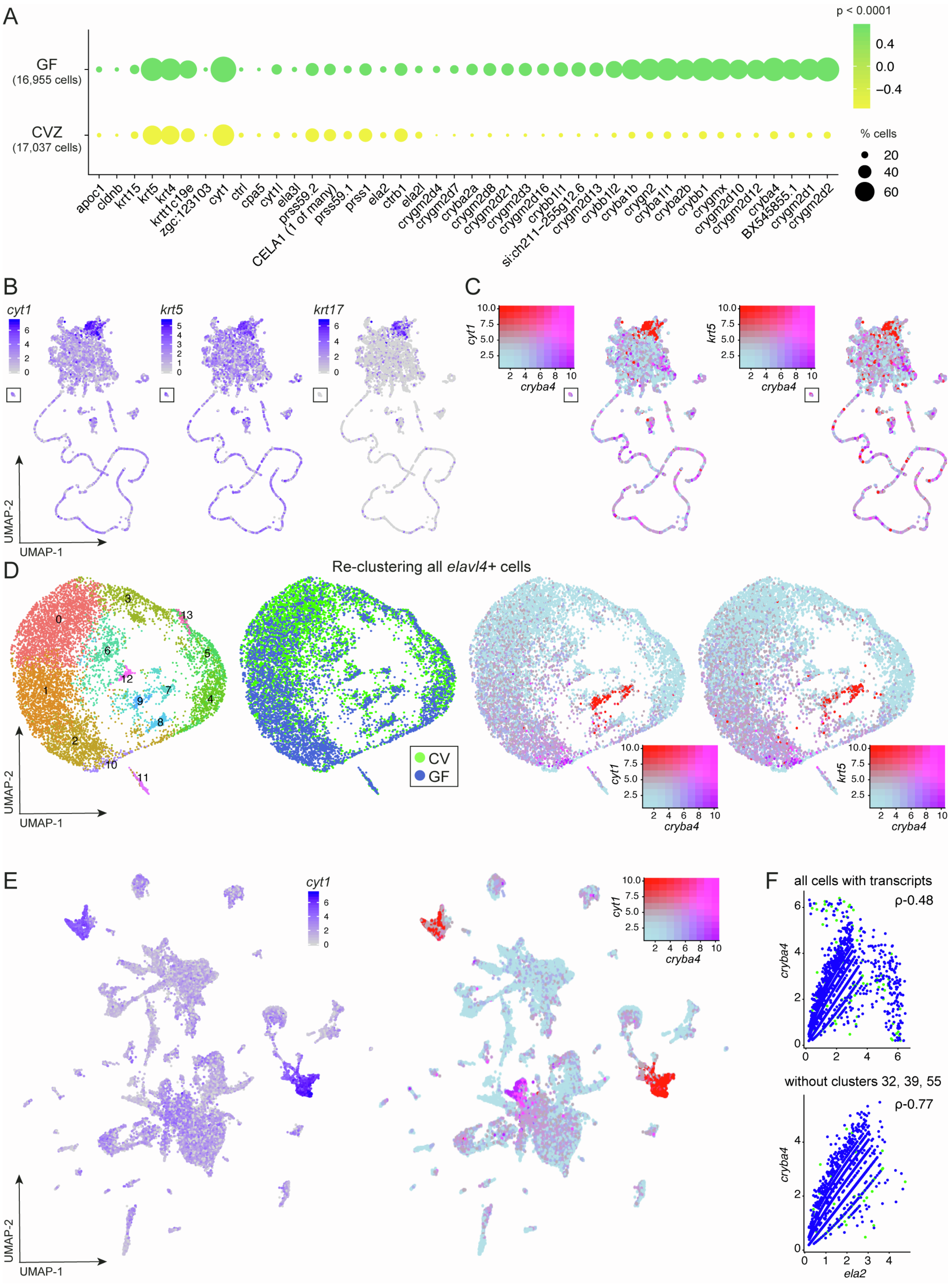

Supplemental Figure 12. Co-expression of Crystallin and Select Keratins are Enriched within GF Cells, Related to Figure 6. A) Dotplot features transcripts significantly enriched within GF cells accounting for all cells in whole larvae. B) uMAP plots from corresponding GF

cells from the original clusters used in Fig.6D clustered based on the expression of crystallin genes show that specific keratins (*cyt* and *krt5*) are broadly expressed across GF cells compared to epidermal specific keratin (*krt17*) that maintains its original cell type identity. C) Corresponding uMAP plots show co-expression of *cyt1* and *krt5* with *cryba4* across GF cells. D) uMAP plots show CVZ and GF *elavl4*+ cells separate based on their experimental group when clustered by the top variable gene expression and that co-expression of *cyt1* and *krt5* with *cryba4* is enriched within GF *elavl4*+ cells. E) uMAP plots show that *cyt1* is co-expressed with *cryba4* across diverse cell types within whole larvae. F) Scatterplots demonstrate the Pearson correlation of *cryba4* and *ela2* across cells in the dataset that express both transcripts and without cells from the exocrine pancreas cluster 32 and lens clusters 39 and 55).
