# Supplementary material for: Cell-type-specific responses to the microbiota across all tissues of the larval zebrafish
Source: Cell Rep. Author manuscript; Available in PMC 2023 Oct 23. (PMC10423310; doi:10.1016/j.celrep.2023.112095)
Supplement: MMC11 [file NIHMS1880944-supplement-MMC11.zip › DataS5/README_S.Figure7_PanelB_DEGs_GOterms.docx]

For files included in the S.Figure7_PanelB_DEGs_GOterms files:

- Each folder represents data for each subcluster represented in S.Figure 7B.
- There are 3 files in each folder:

**subClu#_CVZvGF.tsv:** list of differentially expressed genes (DEGS) between CVZ and GF cells for the given subcluster

- The data listed in this type of labeled spreadsheet shows the original data generated from Seurat FindMarkers function (see Methods). The column names are as follows

**gene:** Ensemble ID

**gene_NAME:** shorthand name of gene used in ZFIN

**p_val_adj:** adjusted p-value for CVZ versus GF comparison

**p_val:** p-value for CVZ versus GF comparison

**avg_log2FC:** average log fold change (base 2)

-positive ave_logFC indicates enrichment within CVZ cells

-negative ave_logFC indicates enrichment within GF cells

**pct.1:** percentage of cells expressing gene within the CVZ cells of the cluster

**pct.2:** percentage of cells expressing gene within the GF cells of the cluster

**GOresults_subClu#_CVZup_fdr.tsv:** list of GO terms generated using the list of DEGs enriched within CVZ cells with a false discovery rate correction (fdr)

**GOresults_subClu#_GFup_fdr.tsv**: list of GO terms generated using the list of DEGs enriched within GF cells with a false discovery rate correction (fdr)

- The data listed in .tsv the prefix ‘GOresults’ shows the original data generated from the ClusterProfiler enrichGO function (see Methods). The column names are as follows and further described <http://geneontology.org/>

**Ontology:** type (molecular function (MF), cellular component (CC), biological process (BP))

**ID:** Gene Ontology ID number

**Description:** description of ontology term

**pvalue:** p-value

**p.adjust:** adjusted p-value using fdr

**qvalue:** adjusted p-value using Benjamini-Hochberg procedure

**geneID:** the individual genes by ENSEMBLE ID that correspond to the GO term

**Count:** number of genes from DEG list that correspond to GO term
