# Supplementary material for: Cell-type-specific responses to the microbiota across all tissues of the larval zebrafish
Source: Cell Rep. Author manuscript; Available in PMC 2023 Oct 23. (PMC10423310; doi:10.1016/j.celrep.2023.112095)
Supplement: MMC13 [file NIHMS1880944-supplement-MMC13.zip › DataS7/README_Figure4_PanelD_GOanalysis.docx]

ImmuneCells_ZebrafishAtlas5dpf_up_versusGF6dpf_GOsorting.xlsx:

- This workbook is a summary of comparing gene expression between the 5dpf immune cells (macrophages/neutrophils) from the Developmental Zebrafish Atlas and the equivalent 6dpf GF immune cells
- This workbook includes 5 tabs:

Tab 1.ZF_Atlas5dpf_*DEGs*: list of genes enriched within the Developmental Zebrafish Atlas 5dpf immune cells versus 6dpf GF immune cells, column names are:

**gene:** Ensemble ID

**gene_NAME:** shorthand name of gene used in ZFIN

**p_val_adj:** adjusted p-value ZF_Atlas 5dpf immune cells versus 6dpf GF immune cells

**p_val:** p-value for ZF_Atlas 5dpf immune cells versus 6dpf GF immune cells

**avg_log2FC:** average log fold change (base 2)

-positive ave_logFC indicates enrichment within ZF_Atlas 5dpf immune cells

-negative ave_logFC indicates enrichment within 6dpf GF immune cells

**pct.1:** percentage of cells expressing gene within cells of ZF_Atlas 5dpf immune cells

**pct.2:** percentage of cells expressing gene within 6dpf GF immune cells

Tab 2. *GOresults_ZF_Atlas5dpf_up*: entire list of GO terms found based on list of DEGs enriched within ZF_Atlas 5dpf immune cells versus 6dpf GF immune cells. Column names are described:

**Ontology:** type (molecular function (MF), cellular component (CC), biological process (BP))

**ID:** Gene Ontology ID number

**Description:** description of ontology term

**pvalue:** p-value

**p.adjust:** adjusted p-value using fdr

**qvalue:** adjusted p-value using Benjamini-Hochberg procedure

**geneID:** the individual genes by ENSEMBLE ID that correspond to the GO term

**Count:** number of genes from DEG list that correspond to GO term

Tab 3. *CategorySorting:* This spreadsheet illustrates how GO terms were binned into larger categories. GOterms binned into the same category have matching colors and the column names are described as above but with some additions:

**(-)Log(p.adjust):** calculated -Log of adjusted p-value

**total gene check:** sum of the ‘Count’ column

**unique genes:** total number of unique genes within the GO term category

**total pathways:** total GO terms binned into larger GO term category

Tab 4. *Table:* This data culminates the categories described in the *CategorySorting* tab 3. Column names are described as above but with some additions:

**TOP p.adjust:** lowest adjusted p-value associated with a GO term/category

**TOP(-)Log(p.adjust):** largest -Log(p.adjust) associated with a GO term/category

**BOTTOM(-)Log(p.adjust):** smallest -Log(p.adjust) associated with a GO term/category

- Horizontal plots illustrate the larger GO categories by the TOP(-)Log(p.adjust) (left) and by the number of unique genes within the category (right)

Tab 5. *PathwaysCharted:* This spreadsheet reformats the GO term ‘Description’ such that the genes (shown by ENSEMBLE IDs) are listed below them. The GO terms are listed left to right in the order that they were categorized in *CategorySorting* Tab 3.
