# Supplementary material for: Cell-type-specific responses to the microbiota across all tissues of the larval zebrafish
Source: Cell Rep. Author manuscript; Available in PMC 2023 Oct 23. (PMC10423310; doi:10.1016/j.celrep.2023.112095)
Supplement: MMC14 [file NIHMS1880944-supplement-MMC14.zip › DataS8/README_Figure5_PanelB_GenesLIsts.docx]

For spreadsheets included in the Figure5_PanelB_GenesLists file:

- Each spreadsheet represents gene expression data for each subcluster generated from the re-clustering of Gnotobiotic Atlas cluster33 as shown in Figure 5B.
- The data listed in each spreadsheet shows the original data generated from Seurat FindConservedMarkers function (see Methods). The column names are as follows

**gene:** Ensemble ID

**gene_NAME:** shorthand name of gene used in ZFIN

**CVZ_p_val_adj:** adjusted p-value for gene expression in CVZ cells of cluster versus CVZ cells outside of cluster

**CVZ_p_val:** p-value for gene expression in CVZ cells of cluster versus CVZ cells outside of cluster

**CVZ_avg_logFC:** average log fold change (base 2) of CVZ in cluster versus CVZ cells outside of cluster

-positive CVZ_ave_logFC indicates enrichment within CVZ cells within cluster

-negative CVZ_ave_logFC indicates enrichment within CVZ cells outside of cluster

**CVZ_pct.1:** percentage of cells expressing gene within the CVZ cells of the cluster

**CVZ_pct.2:** percentage of cells expressing gene within the CVZ cells outside the cluster

**GF_p_val_adj:** adjusted p-value for gene expression in GF cells of cluster versus GF cells outside of cluster

**GF_p_val:** p-value for gene expression in GF cells of cluster versus GF cells outside of cluster

**GF_avg_logFC:** average log fold change (base 2) of GF in cluster versus GF cells outside of cluster

-positive GF_ave_logFC indicates enrichment within GF cells within cluster

-negative GF_ave_logFC indicates enrichment within GF cells outside of cluster

**GF_pct.1:** percentage of cells expressing gene within the GF cells of the cluster

**GF_pct.2:** percentage of cells expressing gene within the GF cells outside the cluster

**max_pval:** largest p-value of p-value calculated by each treatment (CVZ versus GF)

**min_pval:** smallest p-value of p-value calculated by each treatment (CVZ versus GF)
