# Supplementary material for: Cell-type-specific responses to the microbiota across all tissues of the larval zebrafish
Source: Cell Rep. Author manuscript; Available in PMC 2023 Oct 23. (PMC10423310; doi:10.1016/j.celrep.2023.112095)
Supplement: MMC15 [file NIHMS1880944-supplement-MMC15.zip › DataS9/README_Figure5_PanelD_subClu6versus4and5.docx]

Within the Figure5_PanelD_subClu6versus4and5 folder are 3 spreadsheets:

**subClu6v4and5.tsv:** list of differentially expressed genes (DEGS) between subcluster 6 versus 4 and 5. The data listed in this type of labeled spreadsheet shows the original data generated from Seurat FindMarkers function (see Methods). The column names are as follows

**gene:** Ensemble ID

**gene_NAME:** shorthand name of gene used in ZFIN

**p_val_adj:** adjusted p-value for gene expression within subcluster 6 versus subcluster 4 and 5

**p_val:** p-value for gene expression within subcluster 6 versus subcluster 4 and 5

**avg_logFC:** average log fold change (base 2)

-positive ave_logFC indicates enrichment within subcluster 6

-negative ave_logFC indicates enrichment within subcluster 4 and 5

**pct.1:** percentage of cells expressing gene within subcluster 6

**pct.2:** percentage of cells expressing gene subcluster 4 and 5

**GOresults_subClu6up.tsv:** list of GO terms generated using the list of DEGs enriched within subcluster 6 versus 4 and 5. The column names are as follows

**Ontology:** type (molecular function (MF), cellular component (CC), biological process (BP))

**ID:** Gene Ontology ID number

**Description:** description of ontology term

**pvalue:** p-value

**p.adjust:** adjusted p-value using fdr

**qvalue:** adjusted p-value using Benjamini-Hochberg procedure

**geneID:** the individual genes by ENSEMBLE ID that correspond to the GO term

**Count:** number of genes from DEG list that correspond to GO term

**SubCluster6v4and5_GOsorting_subClu6up.tsv:** This workbook illustrates how redundant GO terms were binned into larger GO categories. This workbook includes 4 tabs:

Tab 1. GOresults_subClub6up: list of GO terms as a result of genes enriched within subcluster 6. The column names are:

**Ontology:** type (molecular function (MF), cellular component (CC), biological process (BP))

**ID:** Gene Ontology ID number

**Description:** description of ontology term

**pvalue:** p-value

**p.adjust:** adjusted p-value using fdr

**qvalue:** adjusted p-value using Benjamini-Hochberg procedure

**geneID:** the individual genes by ENSEMBLE ID that correspond to the GO term

**Count:** number of genes from DEG list that correspond to GO term

Tab 2 *CategorySorting:* This spreadsheet illustrates how GO terms were binned into larger categories. GOterms binned into the same category have matching colors and the column names are described as above but with some additions:

**(-)Log(p.adjust):** calculated -Log of adjusted p-value

**total gene check:** sum of the ‘Count’ column

**unique genes:** total number of unique genes within the GO term category

**total pathways:** total GO terms binned into larger GO term category

Tab 3. *Table:* This data culminates the categories described in the *CategorySorting* tab 2. Column names are described as above but with some additions:

**TOP p.adjust:** lowest adjusted p-value associated with a GO term/category

**TOP(-)Log(p.adjust):** largest -Log(p.adjust) associated with a GO term/category

**BOTTOM(-)Log(p.adjust):** smallest -Log(p.adjust) associated with a GO term/category

- Horizontal plots illustrate the larger GO categories by the TOP(-)Log(p.adjust) (left) and by the number of unique genes within the category (right)

Tab 4. *PathwaysCharted:* This spreadsheet reformats the GO term ‘Description’ such that the genes (shown by ENSEMBLE IDs) are listed below them. The GO terms are listed left to right in the order that they were categorized in *CategorySorting* tab 2.
