# Supplementary material for: Cell-type-specific responses to the microbiota across all tissues of the larval zebrafish
Source: Cell Rep. Author manuscript; Available in PMC 2023 Oct 23. (PMC10423310; doi:10.1016/j.celrep.2023.112095)
Supplement: MMC16 [file NIHMS1880944-supplement-MMC16.zip › DataS10/README_Figure5_PanelD_subClu6_CVZvGF.docx]

The spreadsheet subClu6_CVZvGF.tsv: lists the Differentially Expressed Genes (DEG) between CVZ and GF cells within subcluster6 of Figure 5D. The column names are as follows:

- The data listed in each spreadsheet shows the original data generated from Seurat FindMarkers function (see Methods). The column names are as follows

**gene:** Ensemble ID

**gene_NAME:** shorthand name of gene used in ZFIN

**p_val_adj:** adjusted p-value CVZ versus GF cells

**p_val:** p-value for CVZ versus GF cells

**avg_log2FC:** average log fold change (base 2)

-positive ave_logFC indicates enrichment within CVZ cells

-negative ave_logFC indicates enrichment within GF cells

**pct.1:** percentage of cells expressing gene within CVZ cells of subcluster

**pct.2:** percentage of cells expressing gene within GF cells of subcluster
