# Supplementary material for: Cell-type-specific responses to the microbiota across all tissues of the larval zebrafish
Source: Cell Rep. Author manuscript; Available in PMC 2023 Oct 23. (PMC10423310; doi:10.1016/j.celrep.2023.112095)
Supplement: MMC7 [file NIHMS1880944-supplement-MMC7.zip › DataS1/README_DEG_GOterm.docx]

For spreadsheets included in the DEG_GOterm_lists files:

- All analyses and comparisons are between the CVZ and GF whole larvae single cell dissociations
- The sub file names correspond with the data comparing the CVZ and GF cells within the associated cluster from the uMAP plots displayed in Figure 1B
- Within each file for each cluster includes several spreadsheets including:

cluster#_CVZvGF.tsv: list of differentially expressed genes (DEGS) between CVZ and GF cells for the given cluster

clu#_GOresults_CVZup.tsv: list of gene ontology (GO) terms generated using the list of DEGs enriched within CVZ cells

clu#_GOresults_CVZup_fdr.tsv: list of GO terms generated using the list of DEGs enriched within CVZ cells with a false discovery rate correction (fdr)

clu#_GOresults_GFup.tsv: list of GO terms generated using the list of DEGs enriched within GF cells

clu#_GOresults_GFup_fdr.tsv: list of GO terms generated using the list of DEGs enriched within GF cells with fdr

Clu#_GOsorting_CVZup.xlsx: workbook that illustrates how redundant GO terms were binned into larger GO categories within the CVZ cells to consolidate meaningful data

cluster#_CVZvGF.tsv

- The data listed in this type of labeled spreadsheet shows the original data generated from Seurat FindMarkers function (see Methods). The column names are as follows

**gene:** Ensemble ID

**gene_NAME:** shorthand name of gene used in ZFIN

**p_val_adj:** adjusted p-value for CVZ versus GF comparison

**p_val:** p-value for CVZ versus GF comparison

**avg_logFC:** average log fold change (base 2)

-positive ave_logFC indicates enrichment within CVZ cells

-negative ave_logFC indicates enrichment within GF cells

**pct.1:** percentage of cells expressing gene within the CVZ cells of the cluster

**pct.2:** percentage of cells expressing gene within the GF cells of the cluster

clu#_GOresults_X_X.tsv

- The data listed in this type of labeled spreadsheet shows the original data generated from the ClusterProfiler enrichGO function (see Methods). The column names are as follows and further described <http://geneontology.org/>

**Ontology:** type (molecular function (MF), cellular component (CC), biological process (BP))

**ID:** Gene Ontology ID number

**Description:** description of ontology term

**pvalue:** p-value

**p.adjust:** adjusted p-value using fdr

**qvalue:** adjusted p-value using Benjamini-Hochberg procedure

**geneID:** the individual genes by ENSEMBLE ID that correspond to the GO term

**Count:** number of genes from DEG list that correspond to GO term

Clu#_GOsorting_CVZup.xlsx

- The data included in these workbooks illustrate how redundant GO terms were binned into larger categories as shown by horizontal plots throughout the figures.
- NOT ALL clusters included in the DEG_GOterm_lists files will have this workbook
- Each workbook includes 5 tabs:

Tab 1. *CVZ_DEGs*: list of genes enriched within CVZ cells for the cluster, column names are:

**gene:** Ensemble ID

**gene_used_GoTerm:** Ensemble ID if gene was included in GO analysis

**gene_NAME:** shorthand name of gene used in ZFIN

**p_val_adj:** adjusted p-value for CVZ versus GF comparison

**p_val:** p-value for CVZ versus GF comparison

**avg_logFC:** average log fold change (base 2)

-positive ave_logFC indicates enrichment withing CVZ cells

-negative ave_logFC indicates enrichment within GF cells

**%CVZ:** percentage of cells expressing gene within the CVZ cells of the cluster

**%GF:** percentage of cells expressing gene within the GF cells of the cluster

**no.DEGs:** number of DEGs enriched within CVZ cells of the cluster

**no.DEGs:** used in GoTerm: number of DEGs identified/used in GO term analysis

Tab 2. *GOresults_CVZup_fdr*: entire list of GO terms found based on list of DEGs enriched within CVZ cells versus GF for the cluster. Column names are described above.

Tab 3. *CategorySorting:* This spreadsheet illustrates how GO terms were binned into larger categories. GOterms binned into the same category have matching colors and the column names are described as above but with some additions:

**(-)Log(p.adjust):** calculated -Log of adjusted p-value

**total gene check:** sum of the ‘Count’ column

**unique genes:** total number of unique genes within the GO term category

**total pathways:** total GO terms binned into larger GO term category

Tab 4. *Table:* This data culminates the categories described in the *CategorySorting* tab 3. Column names are described as above but with some additions:

**TOP p.adjust:** lowest adjusted p-value associated with a GO term/category

**TOP(-)Log(p.adjust):** largest -Log(p.adjust) associated with a GO term/category

**BOTTOM(-)Log(p.adjust):** smallest -Log(p.adjust) associated with a GO term/category

- Horizontal plots illustrate the larger GO categories by the TOP(-)Log(p.adjust) (left) and by the number of unique genes within the category (right)

Tab 5. *PathwaysCharted:* This spreadsheet reformats the GO term ‘Description’ such that the genes (shown by ENSEMBLE IDs) are listed below them. The GO terms are listed left to right in the order that they were categorized in *CategorySorting* Tab 3.
