# Supplementary material for: Cell-type-specific responses to the microbiota across all tissues of the larval zebrafish
Source: Cell Rep. Author manuscript; Available in PMC 2023 Oct 23. (PMC10423310; doi:10.1016/j.celrep.2023.112095)
Supplement: MMC9 [file NIHMS1880944-supplement-MMC9.zip › DataS3/README_Figure2_PanelC_GenesLIsts.docx]

For spreadsheets included in the Figure2_PanelC_GenesLists file:

- Each spreadsheet represents gene expression data for each cluster in Figure2C Mapping Cluster 45.
- The data listed in each spreadsheet shows the original data generated from Seurat FindMarkers function (see Methods). The column names are as follows

**gene:** Ensemble ID

**ZFIN_name:** shorthand name of gene used in ZFIN

**full_name:** full name of gene used in ZFIN

**p_val_adj:** adjusted p-value for gene expression within the cluster versus outside the cluster

**p_val:** p-value for gene expression within the cluster versus outside the cluster

**avg_logFC:** average log fold change (base 2)

-positive ave_logFC indicates enrichment within the cluster

-negative ave_logFC indicates enrichment outside the cluster

**pct.1:** percentage of cells expressing gene within the cluster

**pct.2:** percentage of cells expressing gene outside of the cluster
