# Supplementary material for: Cell-type-specific responses to the microbiota across all tissues of the larval zebrafish
Source: Cell Rep. Author manuscript; Available in PMC 2023 Oct 23. (PMC10423310; doi:10.1016/j.celrep.2023.112095)
Supplement: MMC24 [file NIHMS1880944-supplement-MMC24.zip › DataS18/README_Figure6_PanelG_GOresults_subcluster.docx]

For spreadsheets included in the Figure6_PanelF_GOresults_subcluster file:

- Each spreadsheet represents the GO results from enriched gene expression per subcluster of Cluster Crystalline Genes.
- The data listed in each spreadsheet shows the original data generated from the ClusterProfiler enrichGO function (see Methods). The column names are as follows and further described <http://geneontology.org/>

**Ontology:** type (molecular function (MF), cellular component (CC), biological process (BP))

**ID:** Gene Ontology ID number

**Description:** description of ontology term

**pvalue:** p-value

**p.adjust:** adjusted p-value using fdr

**qvalue:** adjusted p-value using Benjamini-Hochberg procedure

**geneID:** the individual genes by ENSEMBLE ID that correspond to the GO term

**Count:** number of genes from DEG list that correspond to GO term
